# Gene Expression Profiling in Cells with Enhanced γ-Secretase Activity

**DOI:** 10.1371/journal.pone.0006952

**Published:** 2009-09-18

**Authors:** Alexandra I. Magold, Matthias Cacquevel, Patrick C. Fraering

**Affiliations:** Brain Mind Institute, School of Life Sciences, Swiss Federal Institute of Technology (EPFL), Lausanne, Switzerland; Mental Health Research Institute of Victoria, Australia

## Abstract

**Background:**

Processing by γ-secretase of many type-I membrane protein substrates triggers signaling cascades by releasing intracellular domains (ICDs) that, following nuclear translocation, modulate the transcription of different genes regulating a diverse array of cellular and biological processes. Because the list of γ-secretase substrates is growing quickly and this enzyme is a cancer and Alzheimer's disease therapeutic target, the mapping of γ-secretase activity susceptible gene transcription is important for sharpening our view of specific affected genes, molecular functions and biological pathways.

**Methodology/Principal Findings:**

To identify genes and molecular functions transcriptionally affected by γ-secretase activity, the cellular transcriptomes of Chinese hamster ovary (CHO) cells with enhanced and inhibited γ-secretase activity were analyzed and compared by cDNA microarray. The functional clustering by FatiGO of the 1,981 identified genes revealed over- and under-represented groups with multiple activities and functions. Single genes with the most pronounced transcriptional susceptibility to γ-secretase activity were evaluated by real-time PCR. Among the 21 validated genes, the strikingly decreased transcription of PTPRG and AMN1 and increased transcription of UPP1 potentially support data on cell cycle disturbances relevant to cancer, stem cell and neurodegenerative diseases' research. The mapping of interactions of proteins encoded by the validated genes exclusively relied on evidence-based data and revealed broad effects on Wnt pathway members, including WNT3A and DVL3. Intriguingly, the transcription of TERA, a gene of unknown function, is affected by γ-secretase activity and was significantly altered in the analyzed human Alzheimer's disease brain cortices.

**Conclusions/Significance:**

Investigating the effects of γ-secretase activity on gene transcription has revealed several affected clusters of molecular functions and, more specifically, 21 genes that hold significant potential for a better understanding of the biology of γ-secretase and its roles in cancer and Alzheimer's disease pathology.

## Introduction

**Figure 1:**
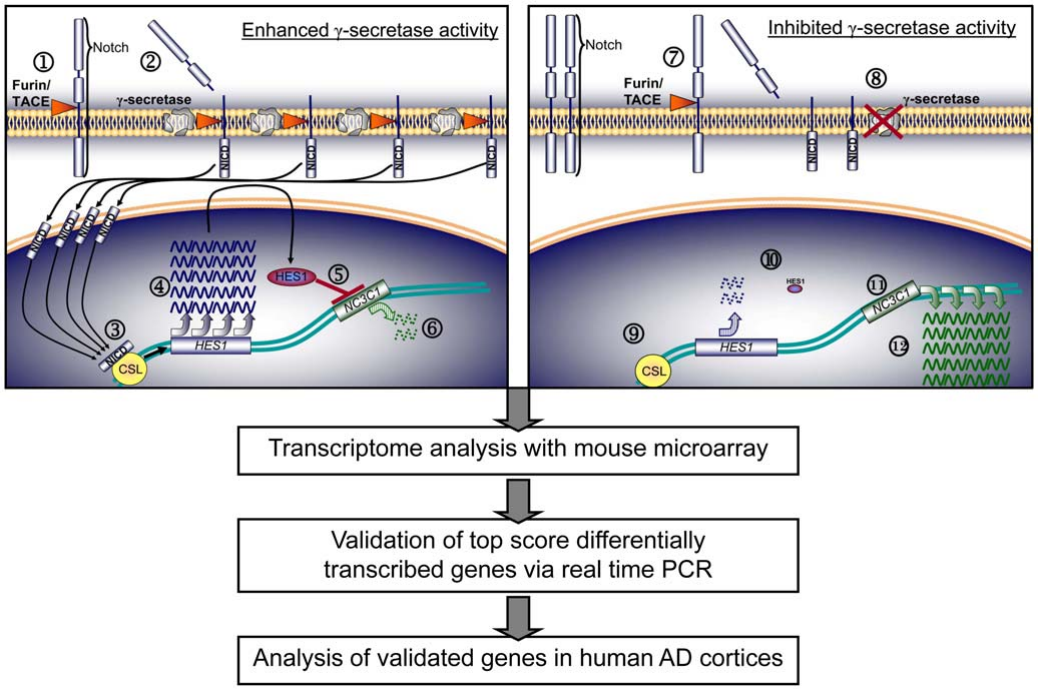
Microarray-based strategy for the identification of genes differentially transcribed in cells with enhanced γ-secretase activity. To identify genes whose transcription is affected by γ-secretase activity, two starkly contrasting conditions were analyzed by cDNA microarray: genetically engineered enhanced γ-secretase (left panel) and pharmacologically inhibited γ-secretase (right panel) in CHO cell lines. For a schematic depiction of the strategy, the Notch-1 receptor signaling pathway is used as an example. After processing by the Furin protease and when activated by binding to its ligands Notch-1 is cleaved at the S2 position by the TACE protease, generating a substrate for γ-secretase (1, 7). Under enhanced (left panel) or inhibited (right panel) γ-secretase activity, the cleavage of the substrate controls the release of the Notch intracellular domain (NICD) (2, 8). With enhanced γ-secretase, increased numbers of NICDs enter the nucleus and interact with CSL (3), leading to the transcription of target genes like Hes1 and Hey (4). The Hes1 transcription repressor inhibits transcription of target genes like NC3C1 (5), with the final consequence being reduced production of NC3C1 mRNA (6). Thus, enhancing γ-secretase leads simultaneously to gene-dependent increase (in the case of Hes/Hey) or decrease (in the case of NC3C1) of mRNA copy numbers. With inhibited γ-secretase, reduced numbers of NICDs (9) lead to the transcription of less Hes1/Hey (10), to reduced inhibition of target genes like NC3C1 (11) and consequently to increased production of NC3C1 mRNA (12). Inhibiting γ-secretase thus leads to gene-dependent decrease (in the case of Hes/Hey genes) or increase (in the case of NC3C1) of mRNA copy numbers. Following mouse cDNA microarray analysis of both transcriptomes, top scoring candidates were evaluated and validated by real time PCR and further analyzed for changes of transcript levels between healthy and AD human brain cortices.

γ-Secretase is an unconventional aspartyl protease (composed of PS1, NCT, Aph-1 and Pen2) with an intramembranous catalytic site that is typical of the class of intramembrane-cleaving proteases (I-CliPs) (for review, see [1], [2]). Via the processing of its substrates and freeing of their intracellular domains (ICDs), γ-secretase regulates a multitude of signaling pathways and biological processes by influencing gene transcription. This is exemplified by the processing of the Notch receptor and the Notch signaling pathway (for a review, see [3]). After specific ectodomain shedding via tumor necrosis factor α converting enzyme (TACE) (Fig. 1, step 1), Notch is further cleaved intramembraneously by γ-secretase (Fig. 1, step 2). The intracellular domain of Notch (NICD) is freed to enter the nucleus, where it interacts with the transcription factor CSL (Fig. 1, step 3). With help from the coactivator Mastermind, CSL is converted from a transcriptional repressor to a transcriptional activator. CSL as an activator leads to the expression of Notch target genes (Fig. 1, step 4), like the Hes or Hey family. Hes1, a transcriptional repressor, inhibits the transcription of NC3C1 (Fig. 1, step 5). Enhanced γ-secretase activity, through its cleavage of Notch, leads to increased transcription of specific genes (Fig. 1, step 4) that repress the expression of other genes (Fig. 1, step 6) to influence a multitude of biological processes. For example, the processing of Notch by γ-secretase is crucial for hepatoblast differentiation [4], epidermis and hair follicle differentiation [5], alveolar differentiation in mammary glands [6], maintenance of skin appendages [7], intestinal stem cell specification [8], induction of satellite cells after injury and maintenance [9] and neural specification of embryonic stem cells [10].

The directions in which γ-secretase activity can up- and down-regulate gene transcription following its cleavage of a variety of substrates is further exemplified by the processing of Amyloid-β (Aβ) precursor protein (APP), one of the better-known γ-secretase substrates. The successive processing of APP by BACE1 and γ-secretase indeed leads to the production of Aβ peptides (a causative agent in the pathogenesis of Alzheimer's disease (AD)), and APP-intracellular domains (AICDs) which, following association with the adaptor protein Fe65 and nuclear translocation, are able to suppress the expression of the major Apolipoprotein ε (ApoE)/lipoprotein receptor LRP1 by binding directly to its promoter [11]. Thus, APP processing is also involved in the regulation of brain ApoE and cholesterol metabolism through LRP1 [11]. As ApoE4 is the major known genetic risk factor for late onset Alzheimer's disease (LOAD) and since AICD production depends on γ-secretase, the latter is implicated in the sporadic form as well. In contrast to LOAD, which correlates directly with age, early onset familial Alzheimer's disease (FAD) is genetic and is mainly caused by mutations in presenilin1 or presenilin2 (PSEN1 or PSEN2), leading to loss of physiological or gain of toxic functions. Murine specific loss of Psen1 in the forebrain has been shown to affect certain aspects of memory [12], [13]. However, it remains difficult to correlate the loss of four murine PSEN alleles with the mild single PSEN allele mutations in FAD [14], [15]. γ-Secretase is thus directly or indirectly implicated in the pathogenesis of both FAD and LOAD, making this protease an attractive therapeutic target for the prevention and/or treatment of AD. γ-Secretase inhibitors/modulators have indeed reached clinical phase III trials [16].

With an increasing number of reports about new γ-secretase substrates and the transcriptional effects of their ICDs being potentially implicated in the pathogenesis of AD or several types of cancer, we see a need for a basic overview of genes and molecular functions that are transcriptionally affected by γ-secretase activity.

## Results

### cDNA microarray analysis of genes differentially transcribed in cells with enhanced γ-secretase activity

In an effort to identify specific alterations of gene transcription as a result of γ-secretase activity, the transcriptomes of two CHO cell lines (biological triplicates were used in each case) with enhanced and inhibited γ-secretase activity were analyzed and compared (strategy depicted in Fig. 1–exemplified by Notch processing). The S-1 cell line overexpresses the four components of γ-secretase (NCT, Aph1a, PS1 and Pen2) and was characterized by a marked increase in the level of PS1 heterodimers and an associated 8-fold increase in γ-secretase activity compared to untransfected controls [17]. The other cell line consisted of the original parental wild type CHO cells incubated with DAPT, a well-known γ-secretase inhibitor. We strategically chose those two conditions, overexpression of γ-secretase and inhibition of its activity, to amplify the activity-dependent effects on gene transcription levels (i.e., amplification of the signal from the cDNA microarray). To reduce potential effects due to changes in the protein levels of the γ-secretase subunits as opposed to changes in its activity that we are interested in, we used chemical inhibition (DAPT) of γ-secretase activity instead of gene silencing, which ultimately leads to changes in protein levels [18]. Biological functions have indeed been reported mainly for the γ-secretase subunit PS1, independently to the γ-secretase activity. However, because treatment of CHO cells with DAPT has been recently reported to exacerbate the secretion of exosomes [19], we cannot exclude at this stage that some detected genes may be exosome-related in response to the DAPT treatment. The gene transcription levels of the two cell lines were analyzed using a mouse cDNA microarray (Fig. 1 and Material and Methods) [20] because of the absence of a readily available DNA microarray based upon hamster gene sequences or cDNA clones. This lack has been noticed, and cross-species reactivity of a mouse microarray hybridized with CHO-derived samples has been investigated recently by De Leon Gatti et al. [21]. This group generated an EST-based CHO microarray and compared it with results from a mouse microarray and vice versa. They state that cross-species hybridization yielded 89.6% overlap in their arrays, non-contradicting results and led only to a decrease in sensitivity resulting in detection of fewer differentially expressed genes. Accordingly, we probably have not detected all differentially expressed genes, but we have detected a significant amount, including clusters of functional relevance. For example, Neprilysin, an Aβ-degrading enzyme of functional relevance to AD that has been previously shown to be transcriptionally downregulated in PSEN1/PSEN2 double knock out fibroblasts and to exhibit reduced activity under chemical (DAPT) inhibition of γ-secretase in mouse neurons [22], was not detected in the current study. Collectively, this supports the use of CHO cells with a mouse microarray. The microarray data set discussed in this publication has been deposited in the NCBI Gene Expression Omnibus (GEO, http://www.ncbi.nlm.nih.gov/geo/) and is accessible through GEO Series accession number GSE16379.

The mouse microarray consistently detected the four human γ-secretase subunits overexpressed in the S-1 cell line (Table 1). By applying a cut-off based on the false discovery rate (FDR, i.e., the probability to wrongly accept a difference between the two conditions) with a p value of 0.005, we found 2658 EST clones (1981 genes) to be differentially expressed, with 1241 EST clones of increased and 1417 EST clones of decreased transcription upon enhanced γ-secretase activity (Supplemental Material, Dataset S1 and Dataset S2).

**Table 1:** Mouse microarray detection of γ-secretase components overexpressed in CHO cells with enhanced γ-secretase activity.

Gene	Protein	Probe ID	FC	adj.P.Val.
PSENEN	presenilin enhancer 2 homolog (Pen2)	H3153E12	17.1	2.01E-0.4
APH1A	anterior pharynx defective 1 homolog A (Aph1a)	H3009H07	8.1	2.29E-04
PSEN1	presenilin 1 (PS1)	H3150D02	4.3	5.07E-04
NCSTN	nicastrin (NCT)	H3012F08	2.6	3.48E-02

Gene and Protein names are displayed in first and second column from the left; Probe IDs, fold change (FC) and adjusted P Value (for false discovery rate) follow in columns 3, 4 and 5.

### Functional clustering of genes differentially transcribed in cells with enhanced γ-secretase

**Figure 2:**
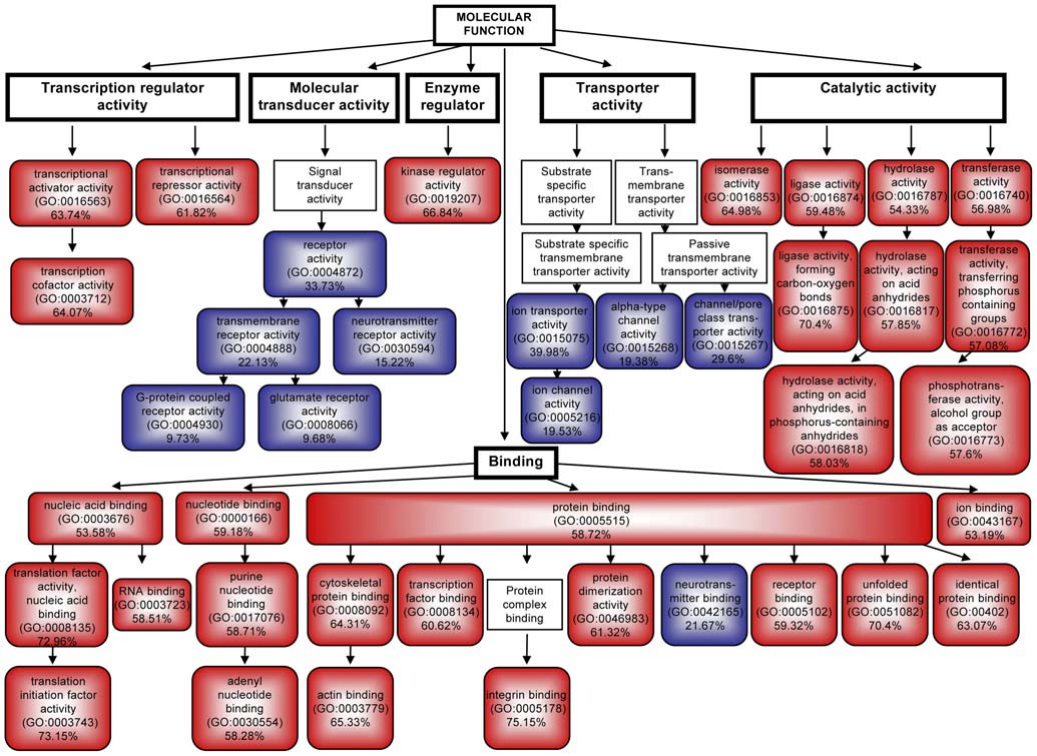
Functional clustering of differentially transcribed genes in cells with enhanced γ-secretase activity. Categories within the Molecular Function GO hierarchy that were over- and under- represented among the genes that were differentially transcribed in cells with enhanced γ-secretase activity. Red boxes display GO terms that were overrepresented; blue boxes indicate GO terms that were under-represented. Black boxes represent main molecular functional clusters and arrows point toward according subclusters. The clustering of 1981 differentially transcribed genes was performed with DAVID and the FatiGo tool [23].

Mapping clusters of genes of GO functions transcriptionally susceptible to γ-secretase activity levels resulted in a GO hierarchy-dependent tree that will provide further orientation for γ-secretase research. Functional clustering of 2658 differentially expressed sequences (1981 genes, Supplemental Material, Dataset S3) was performed using the FatiGo tool [23]. Comparing the representation of functional groups of genes throughout the entire mouse genome with their representation within the group of differentially transcribed genes allowed us to see whether clusters of genes of a specific functional group were enriched in the differentially expressed set. Clusters of over- and underrepresented genes were detected (Fig. 2). The gene functions “transcription regulator activity”, “kinase regulator activity”, “catalytic activity” and “binding ” were found to be overrepresented among the 2658 sequences (1981 genes) that were differentially transcribed. The cluster of “molecular transducer activity”, through its subclusters in the GO hierarchy: “receptor activity” GO0004872, “transmembrane receptor activity” GO 0004888 and “neurotransmitter receptor activity” GO0030594, as well as the cluster of “transporter activity”, via its subcluster of “ion transporter activity” GO0015075, were underrepresented (Fig. 2, blue boxes). This is significant since neurotransmitter activity and transmembrane receptors are well within the focus of current AD research [24].

Supporting our hypothesis that γ-secretase has a role in multiple transcriptional regulatory activities, the GO cluster of “transcription regulator activity” is overrepresented through both its subclusters “transcriptional activator activity” GO0016563 and “transcriptional repressor activity” GO 0016564 (Fig. 2, red boxes. Single member genes of each cluster are annotated in Supplemental Material Dataset S3). A well-described gene within the activator cluster is β-catenin (CTNNB1, FC = 3, p = 0.001), whereas an example of a gene in the cluster of “transcriptional repressor activity” is HES1. Hes1 (FC = 5.4, p = 7.69E-04) is a transcription factor that has previously been reported as a downstream target of the Notch signaling pathway [25] (Fig. 1). Like the examples above, 56 other transcription-related genes were found to be differentially transcribed with enhanced γ-secretase activity (Supplemental Material, Dataset S4). Consistent with these findings, several known substrates of the enzyme were detected on the microarray as well (Table 2). This suggests a possible feedback mechanism by which the augmented processing of these substrates by γ-secretase might lead to their altered transcription. The overrepresentation of genes in the clusters of enzymatic activity, such as “kinase regulator activity” GO0019207 and “catalytic activity”, through four distinct GO subclusters (“isomerase activity” GO 0016853, “ligase activity” GO0016874, “hydrolase activity” GO0016787 and “transferase activity” GO 00167740–Fig. 2, red boxes), is broad in terms of the type of enzymatic activity and further shows the diversification of the downstream effects of enhanced γ-secretase activity.

**Table 2:** γ-Secretase substrates as reported for differential expression by microarray.

Gene	ProbeID	FC	adj.P.Val.
LRP2	P869	8	7.48E-04
JAG2	N734	7	4.30E-03
APP*	H3132G02	7	1.11E-03
NOTCH3	N732	5	2.78E-03
CDH1	H3050H02	−3	4.26E-03
CD44	H3012H07	−3	1.33E-03
EPHA4	H3122H01	−3	4.82E-03
APLP2	H3154H04	−4	2.22E-03
LRP1	H3105A05	−5	2.75E-04

Lrp2, Jag2, App, Notch, Cdh1, CD44, Aplp2 and Lrp1 are known γ-secretase substrates and were found to have altered gene expression with enhanced γ-secretase activity.

* overexpressed in S-1 cells.

The most complex cluster of molecular function that is overrepresented among the differentially transcribed genes identified in our microarray analysis is the GO function termed “Binding”. This cluster is overrepresented through six subclusters and several subclusters of these (Fig. 2, lower part). Consistent with transcription regulation, the binding subclusters of “nucleic acid binding” GO0003676 and “nucleotide binding” GO0000166 are overrepresented. The cluster of “ion binding” GO0043167 is overrepresented as well as the cluster of “protein binding” GO0005515. A consistently overrepresented subcluster of the latter is “cytoskeletal protein binding” GO0008092 (Fig. 2). Cytoskeletal proteins have long been known to play a role in AD and Tauopathies. They are targets of the cell polarity Wnt pathway, and their dynamics have recently been shown to be affected by AICD [26].

“Receptor binding” GO0005102 also includes the Notch ligand and known γ-secretase substrate Jagged 2 [27], [28], as well as the α-secretase ADAM 10 [29], four members of the Wnt family (Wnt6, 7a, 9b and 10a) and, the aforementioned β-catenin. Indeed, the translocation of β-catenin is mediated by ADAM 10, which is of the same functional cluster [30].

By clustering transcriptionally affected genes, we demonstrate that neurotransmitter, transcription regulator and enzymatic activities, transmembrane receptor and cytoskeletal proteins functional groups are affected by γ-secretase activity in their mRNA copy numbers.

### Validation of differential gene transcription by quantitative real-time PCR

**Figure 3:**
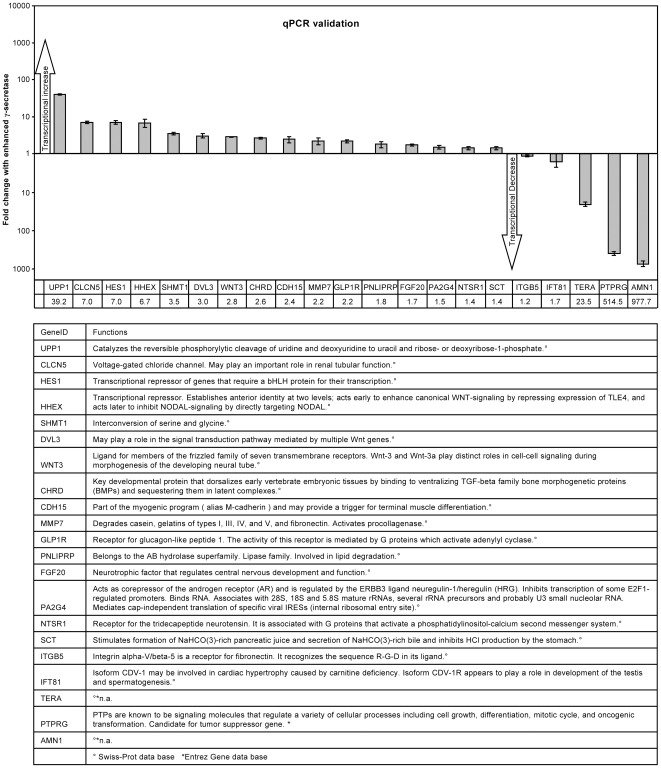
Real time PCR validation of differentially transcribed genes in cells with enhanced γ-secretase activity. Fifty of the top scoring genes identified by microarray analysis to be differentially transcribed with enhanced γ-secretase activity were analyzed by real time PCR with primers based on mouse gene sequences. These primers showed specific and reproducible amplification for 35 genes and a total of 21 genes were validated to be differentially transcribed with enhanced γ-secretase activity. Relative quantification is expressed as fold change of transcript levels compared to inhibited γ-secretase conditions. Fold difference is displayed on the Y-axis and in table below X-axis. Error bars reflect standard deviations of biological triplicates.

For specific analysis of single genes, the fifty most prominently transcriptionally altered genes were evaluated by real time PCR. Mouse code based primers worked reproducibly and specifically for 35 genes. Among them, 21 genes were found to be differentially transcribed with enhanced γ-secretase activity (Fig. 3 upper panel, annotations lower panel). The highest increase in transcription levels was detected for UPP1, a gene encoding an enzyme (Uridine phosphorylase, UPase) directly implicated in the processing of uridine. UPP1 was confirmed by real time PCR to have a 39.2-fold increase in transcription levels (Fig. 3 upper panel). Uridine is a strong sleep-promoter and is crucial for RNA, DNA and membrane biosynthesis [31]. Because of the latter, a lack of uridine (caused by increased UPase) would thus first damage cells with a large membrane to cytoplasm ratio, one of the most extreme ratios being found in neurons due to their axon and dendrite structure [32]. Interestingly oral administration of uridine has improved AD phenotypes [33], [34]. The protein Upp1 also interacts with Vimentin [35], the distribution of which is characteristically altered in FAD fibroblasts [36].

The Notch-dependent transcriptional repressor Hes1 was also confirmed by real time PCR with a 7-fold increase in mRNA levels under enhanced γ-secretase activity (Fig. 3).

Importantly, we found several key players of the three Wnt pathways to be transcriptionally altered in response to enhanced γ-secretase. We confirmed one of these, Wnt3a, to be increased by 2.8-fold in S-1 cells (Fig. 3). Aoyama et al. have reported that Wnt3a can influence Notch protein levels and increase Notch1 activation [37], which increases the effect of enhanced γ-secretase even further through substrate enhancement. Thus, enhanced γ-secretase activity may lead to increased WNT3A transcription, which in turn can increase the protein levels of the NICD-carrying γ-secretase substrate. This proposed enhancement of the canonical Wnt pathway is further supported by the recent observation that β-catenin (the central protein that also ties PS1 to the pathway) modulates the level and transcriptional activity of Notch1/NICD through their direct interaction [38]. Several proteins, including Frizzled and Disheveled (Dvl), relay the Wnt signal along the canonical pathway between Wnt and β-catenin. We found that they both show increased gene transcription in our microarray analysis. DVL3 was confirmed by qPCR to increase in mRNA copy numbers by 3-fold (Fig. 3). Taken together with the microarray data showing differential transcription of PROC, DKK, LRP5/6, GBP, AXIN, β-catenin, C-JUN and CYC D (all part of the canonical Wnt pathway–for further details see ‘Discussion’), our data suggest a strong transcriptional effect on this pathway by γ-secretase activity and resulting alterations in gene expression. CYC D has also been reported to be downregulated by Protein tyrosine phosphatase receptor type G (PTPRG) [39]. We confirmed by real time PCR that PTPRG transcript level is reduced by 515-fold (Fig. 3). AMN1 (levels down 978-fold) has also been connected with cell cycle regulation in yeast, but its role in mammals is not well known. TERA, a gene of unknown function (levels down 24-fold), has also been associated with Wnt antagonism (see Fig. 3 and results of human cortex analysis). β-actin served as housekeeping gene.

### Protein interaction data suggest Wnt pathways as a major target of γ-secretase susceptible gene transcription

**Figure 4:**
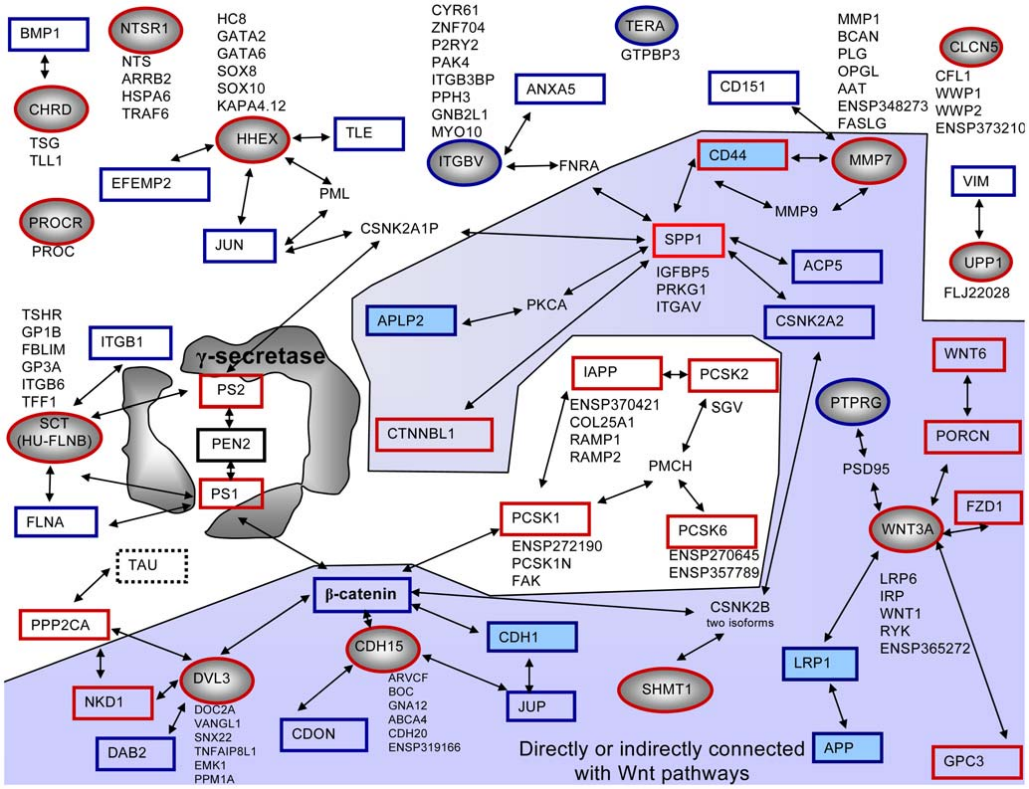
Protein-protein interaction network of proteins encoded by genes differentially transcribed in cells with enhanced γ-secretase activity. All interaction partners as reported by experiment based evidence in the string 8.0 database are shown in black and indicated by two headed arrows. Proteins encoded by PCR-validated genes are represented in circles (blue circles for genes of decreased transcription, red circles for genes of increased transcription). Interaction partners encoded by genes identified in our microarray, but having not yet been validated are displayed in quadrangles (blue quadrangles for genes of decreased transcription, red quadrangles for genes of increased transcription). Proteins with blue background are known γ-secretase substrates. The central grey box indicates γ-secretase subunits. Proteins acting directly or indirectly on, or interacting with Wnt pathways are highlighted by a light purple background figure.

In order to see whether γ-secretase affects the transcription of genes encoding interacting proteins, an interaction map of encoded proteins was generated with the string 8.0 data bank exclusively relying on evidence-based data. Clusters of protein interactions suggest the Wnt signaling pathways as a major focus of γ-secretase-affected candidates (Fig. 4, highlighted in grey). Indeed, we found several members of the canonical Wnt pathway, but also some interactors of the planar cell polarity (PCP) pathway and the Wnt/Ca2+ pathway, to have γ-secretase activity susceptible gene transcription (Fig. 4). Some of these genes have been confirmed by real time PCR as well as DIGE experiments (Egger et al., *unpublished*). The largest decrease in gene transcription occurred for the gene encoding the protein Ptprg. This single-pass type I membrane protein dephosphorylates protein tyrosine phosphate and was recently suggested as a candidate tumor suppressor gene in nasopharyngeal carcinoma [39]. The same group reported functional evidence for a critical interaction of Ptprg with the extracellular matrix, which induces cell arrest, changes in cell cycle status and downregulation of cyclin D1 [39]. The latter is strongly affected by the canonical Wnt pathway. Ptprzeta and beta, structurally similar to Ptprg, interact with Psd95 [40], which directly interacts with Wnt3a [41]. We could confirm that WNT3A transcripts show an increase of 2.8-fold (Fig. 3). Further, Wnt3a has also been reported to interact directly with LRP1 (Fig. 4, lower right), a stimulator of the Wnt5a signaling pathway [42] and a known γ-secretase substrate tying γ-secretase to a major AD risk factor, ApoE [43]. Porcn, another protein that interacts with Wnt3a [44], shows a three-fold increase in transcript level by the microarray experiment. Porcn also interacts with Wnt 6 (4-fold increase in microarray) as reported by the same group and is the first player of the canonical Wnt pathway as displayed by the Kegg database (mmu04310, http://www.genome.jp/dbget-bin/show_pathway?mmu04310). Wnt3a interacts with Frizzled 1 [45], which showed a 5-fold increase in mRNA levels by our microarray. Following the canonical Wnt pathway, the first intracellular protein of the Wnt signaling cascade is “Disheveled”. As confirmed by real time PCR, DVL3 mRNA is increased by 3-fold with enhanced γ-secretase activity. Next, with the help of Gbp (microarray reports a 4-fold increase of Gbp2), Gsk-3β is inhibited, which in turn inhibits β-catenin. As made apparent by the graphical overview of interacting proteins encoded by genes we found to be transcriptionally susceptible to γ-secretase activity, β-catenin plays a central role, linking different proteins involved in different Wnt pathways (Fig. 4). Furthermore, β-catenin has been found to function as a major node connecting PS1 and several proteins that are encoded by genes that we found to be differentially transcribed (Fig. 4). β-catenin transcription was shown by the microarray to be 3-fold decreased. It interacts directly with cdh15 (which showed a 2.4-fold increase in transcript levels as confirmed by real time PCR, Fig. 3), with Cdh1 (a known γ-secretase substrate [46]), with PS1 (the γ-secretase catalytic subunit) and other proteins encoded by candidate genes reported by the microarray. In the context of the canonical Wnt pathway, β-catenin affects c-myc (CMYBP 3-fold increased on the microarray), c-jun (3-fold decreased on the microarray) and cyclin D (4-fold decreased on the microarray) - the latter, as mentioned, is also downregulated by Ptprg. Dvl3 however also interacts with other proteins encoded by candidate genes, among which Nkd1 is of special interest since it links the canonical with the planar cell polarity pathway where it has a different effect on Dvl. The Planar cell polarity pathway through several players, among them Rac (3-fold decreased on the microarray) affects gene transcription, as we hypothesize for γ-secretase activity changes. Through a chain of different mediators, the planar cell polarity Wnt pathway affects the cytoskeleton. Our microarray has reported some of these mediators to be differentially transcribed as γ-secretase activity is enhanced; RhoA transcript levels for example are 3-fold decreased. Rock, which is known to directly interact with the γ-secretase substrate CD44 (CD44/Rho Family GTPase/ROCK2) [47], [48], is transcriptionally affected too. Our top candidate UPP1 has only one interaction partner that was also reported to be differentially expressed by the microarray, the cytoskeleton protein vimentin (Vim) [35]. Vimentin itself is not new to AD research, as altered Vim distribution patterns were observed in FAD fibroblasts [36]. Also, UPP1 transcription is regulated by the transcription factor Oct3/4, as is the transcription of another candidate, called SPP1 [49]. Spp1 is a direct interaction partner of the aforementioned γ-secretase substrate CD44 and strongly affects Ca^2+^ levels [50]. It directly interacts with several proteins encoded by candidate genes, including PKCA, which itself directly interacts with Aplp2, a well-known γ-secretase substrate, and Csnk2b, which directly interacts with β-catenin, thus closing the circle. Csnk2b also directly interacts with Shmt1, which has enhanced transcription of 3.5-fold (Fig. 3), and has been further confirmed in DIGE experiments (Egger et al., *unpublished*). The third Wnt pathway mentioned is the Wnt/Ca^2+^ pathway which includes, among others, Plc (Plcb1 5-fold increase on the microarray), CaMKII (4-fold decrease on the microarray) and Calpain (3-fold decrease on the microarray).

Our mapping of genes differentially transcribed with γ-secretase activity shows that they encode proteins that directly interact with each other, with many of them being members of Wnt pathways.

### TERA gene transcription is significantly altered in Alzheimer's disease cortices

Our modeling of extreme levels of γ-secretase activity in CHO cells has revealed γ-secretase-dependent differences in transcript levels of specific genes. One of the major known risk factors for developing Alzheimer's disease is carrying the ApoE4 allele. Recently it was shown that ApoE through LRP1 regulation is connected with γ-secretase [43], which supports the hypothesis of a potential role of γ-secretase in sporadic AD. γ-Secretase is also directly implicated in the inheritable familial early onset forms of AD (FAD), as most cases are caused by mutations in PSEN1, the gene encoding for PS1, the catalytic center of this enzyme.

**Figure 5:**
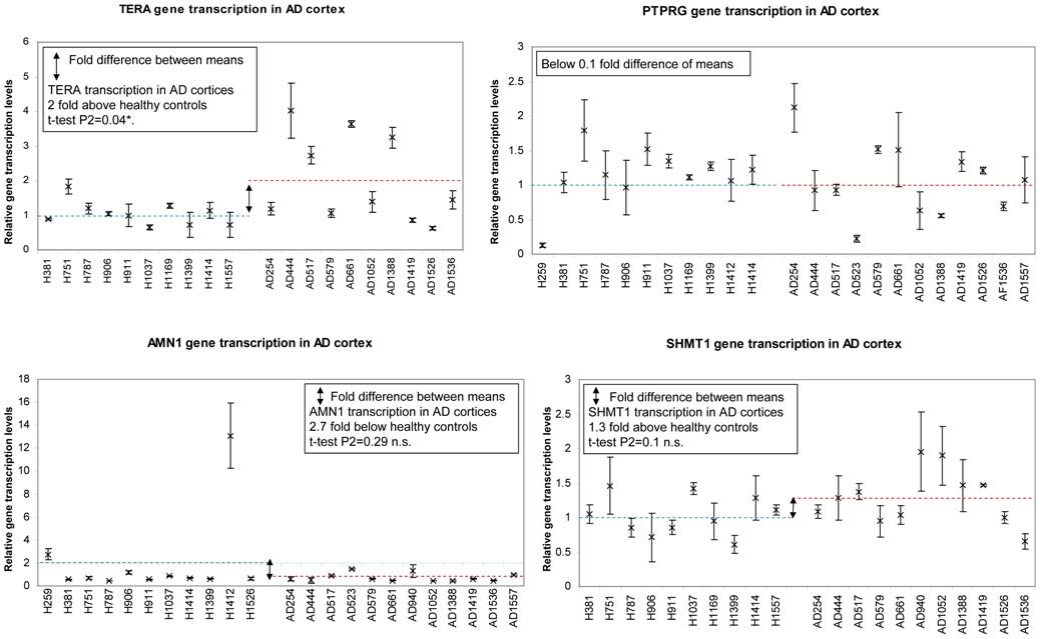
Selected relative gene transcript levels in AD cortices. Real time PCR validated genes differentially transcribed in cells with enhanced γ-secretase activity were selected and their gene transcript levels analyzed in ten to twelve AD and healthy human cortical brain tissue samples. Only the transcript levels of TERA, a gene of unknown function, is significantly altered with a two-fold increase in AD cortices. Note that TERA transcript levels were significantly reduced in cells with enhanced γ-secretase activity (Fig. 3). Relative quantification of gene transcription in CHO cells as well as in brain tissue used β-actin as housekeeping gene. Healthy control levels are displayed on the left part of each diagram, AD transcript levels on the right. Dashed lines indicate mean values for healthy controls (green) and AD cases (red). Double-headed arrows indicate tendencies of differences between groups. P2 values obtained from t-test are indicated in black boxes of the upper part of each diagram.

To investigate whether changes in gene transcription that coincide with alterations of γ-secretase activity levels also differ between sporadic Alzheimer's and healthy human brain tissue, we evaluated our top scoring γ-secretase affected genes in human AD and healthy cortices. Based on β-actin as housekeeping gene, we found one γ-secretase affected gene, TERA, to be significantly differentially transcribed in the AD brain relative to the normal brain. Real-time PCR results showed an average two-fold increased TERA transcript levels (P2 = 0.04) in human AD cortices compared to healthy controls (Fig. 5).

Altogether, the Wnt antagonism gene TERA represents a new candidate for differential expression with γ-secretase activity as well as in AD brain cortex tissue. Whether it is implicated in the pathogenesis of AD requires further investigation.

## Discussion

**Figure 6:**
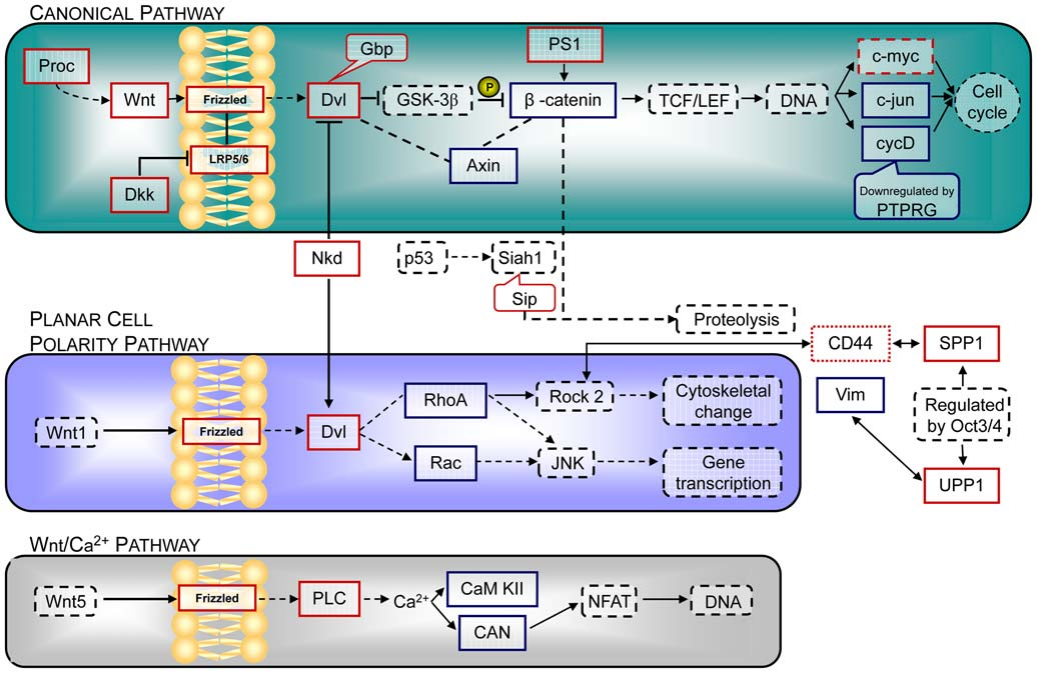
Involvement of γ-secretase-dependently transcribed genes in Wnt pathways. Several key players of the canonical Wnt pathway (green panel) were reported by our microarray to increase (red quadrangles) or decrease (blue quadrangles) in transcript levels under conditions of enhanced γ-secretase activity compared to inhibited activity. β-Catenin is a central node connecting Wnt–Frizzled–Dishevelled to a downstream effect influencing the cell cycle (see also Fig. 4 and interactions of encoded proteins). For better understanding, selected genes that were not detected by the microarray are displayed as well (dashed lines black quadrangles). CycD was reported to be regulated by PTPRG, one of the top scoring candidates for γ-secretase affected gene transcription. Nkd, which we found to be increased in transcript levels, connects the canonical Wnt pathway with the planar cell polarity pathway (blue panel). CD44, a well-known γ-secretase substrate, interacts with SPP1. SPP1 and UPP1, two strong candidates are both under the control of the same transcription factor Oct3/4, as has been suggested for TERA [92]. UPP1 directly interacts with Vimentin (see also Fig. 4), a known player in AD and a cytoskeletal protein. Crucial genes of the Wnt/Ca^2+^ pathway (grey panel) were also found to be differentially expressed in our array. All together, γ-secretase activity influences the transcript levels of genes of the canonical, the planar cell polarity and Ca^2+^ Wnt pathways.

Since the discovery of the roles for NICDs and AICDs in gene transcription, the notion of γ-secretase as a major player in pathologically altered gene transcription patterns has been steadily gaining ground with new substrates and their transcriptionally active ICDs being identified regularly. To investigate the impact of γ-secretase activity on gene transcription, we compared two starkly contrasting situations: genetically engineered enhanced human γ-secretase activity and pharmacologically inhibited γ-secretase activity in CHO cell lines. By investigating the effects of enhanced γ-secretase activity on gene transcription using cDNA microarray analysis, we could show that the canonical, the planar cell polarity (PCP) and the Ca^2+^/Wnt pathways are transcriptionally affected through more than a dozen of Wnt signaling players (summarized in Fig. 6). From Proc and Wnt outside the membrane, through Frizzled and Dvl, to β-catenin and down to cell cycle regulating genes, the canonical Wnt pathway is the most affected of Wnt pathways. Several genes of the PCP Wnt pathway as well as Ca^2+^/Wnt pathways were found to be differentially expressed too (Fig. 6). One of the cell cycle regulating genes is CYC-D, which itself is regulated by one of the most γ-secretase-dependently altered genes reported by us, PTPRG.

Functional clustering of the microarray data revealed the overrepresentation of the “receptor binding” cluster, which includes four different Wnt signaling molecules and β-catenin. β-Catenin also finds itself in the center of interactions of proteins encoded by strongly differentially expressed genes. Components downstream of the canonical Wnt pathway, like c-myc, c-jun and cycD, influence the cell cycle, the latter as mentioned is downregulated by protein tyrosine phosphatase receptor type γ (Ptprg). Interestingly, we found PTPRG transcription to be strongly decreased in cells with enhanced γ-secretase. Barnea et al. [51] identified a subfamily of PTPRs, defined by the carbonic anhydrase-like domain in the extracellular region of PTPRG, and described its expression during hippocampal formation, and in septal and midline thalamic nuclei in the cortex of newborn rats (in contrast to the expression pattern in adult rats, which is reduced to the hippocampal formation). Several groups have shown a connection between alterations in receptor tyrosine phosphatases' expression levels and γ-secretase [52], [53]. However, we report here for the first time, to our knowledge, the transcriptional connection between the receptor tyrosine phosphatase type gamma and γ-secretase.

TERA, a gene that we found to be decreased in transcription (down by 23.5-fold), has been connected to brain development and Wnt antagonism as well. TERA is decreased to minimal transcript levels with enhanced γ-secretase activity (Fig. 3). This gene, encoding a phosphoprotein of unknown function, is upregulated in squamous cell carcinoma (SCC), adenocarcinoma (AC), and colon, ovary, rectum and stomach tumors [54] (suggesting associations with Notch?). It has also been reported that TERA gene expression is increased in day 13 embryonic (E13) and decreased in E17 cortex and maintains low, but consistent expression levels in the subventricular zone (SVZ) [55]. The expression pattern in earlier rather than later stages of brain development and in the location of neuronal stem cell niches, like the SVZ, suggest possible roles for Tera in regenerative processes and raise questions about its function if the gene is being shut down in degenerative disorders like AD [55]. Tera expression has further been found to be maintained in neural progenitors and downregulated during non-neural differentiation, and was shown to have appreciable expression in embryonic stem cells in a screen for functional genes in ES cells that implicated Wnt antagonism in neural differentiation [56].

TERA and the anti-mitotic exit network antagonist 1 (AMN1) map to chromosome 12p11, which is interesting when considering the fact that chromosome 12 has been discussed to contain an unknown LOAD locus for over a decade, and in a recent study including 492 LOAD cases [57]–[59]. In our study, AMN1 transcription is decreased by 978-fold with enhanced γ-secretase activity. The function of AMN1 is not known. However, several expression pattern based studies suggest it functions as a cilia gene in sensory neurons [60]. Another typical cilia gene is intraflagellar transport protein 81 (IFT81) which, among a dozen of known cilia genes, was also shown by the microarray to be differentially expressed with altered γ-secretase activity (see also Fig. 3). More and more evidence has been emerging over the last years that primary cilia, in parallel to their well-established functions in sight, smell and mechanosensation, are key participants in intercellular signaling [61]. The importance of monocilia for the regeneration of olfactory neurons has only been better understood recently [62]. Subventricular zone (SVZ) astrocytes, providing glia as well as neurons for the mammalian olfactory bulb, have primary cilia [63]. They give rise to type C cells, which in turn generate neuroblasts [64] that migrate in the adult brain from the SVZ to the olfactory bulb along the cerebrospinal fluid (CSF) flow. The CSF flow depends on the beating of the ependymal cilia [65]. Cilia genes are not only relevant to the maintenance of adult regeneration in the brain since they uphold the constant flow of the CSF, but also because they are directly implicated in cell cycle control. Polycystins, for example, control the cell cycle through three major pathways with one depending directly on β-catenin [66]. A study of inversin has further shown that flow shear stress as sensed through cilia may regulate the Wnt signaling pathway through β-catenin [67], [68]. Given that fluid flow is crucial for the transport of neuroblasts in the SVZ, one could expect that β-catenin and the Wnt signaling pathway that connects our candidates are also functionally relevant to the cilia genes found in this study. We found both genes of unknown function TERA and AMN1 to be decreased in transcription with enhanced γ-secretase activity. TERA and AMN1 can be connected to neural stem cells through several types of cancer, neural differentiation (in the case of TERA) and through the role of monocilia for neurogenesis (in the case of AMN1). All in all, we have demonstrated that AMN1 and TERA are genes of basically unknown function that are worthy of further investigation to understand their roles in neurogenesis, cancer and γ-secretase biology.

We further report here that UPP1 transcript levels are increased with enhanced γ-secretase activity (by 39.2-fold). UPP1 encodes for uridine phosphorylase (UPase), an enzyme that catalyzes the reversible phosphorylytic cleavage of uridine and deoxyuridine to uracil and ribose- or deoxyribose-1-phosphate [69]. UPP1 expression has been extensively connected to cancer, stem cells and inflammation such as multiple sclerosis [70]–[77]. UPase is induced by vitamin D3 and a mixture of inflammatory cytokines, Interferon gamma, TNF-alpha and IL-1, with the latter two being upregulators of Ptprg [78]. Increased UPP1 transcript levels, associated with enhanced UPase activity cleaving uridine, would potentially have inhibitory effects on several pathways downstream of uridine, like RNA/DNA and membrane synthesis, as well as protein glycosylation, which would in turn trigger long-term neurodegeneration. Particularly, decreased membrane synthesis, in the case of synaptic membranes, would also reduce synaptic activity and plasticity. In support of that, TNF-α and IL-1, inducers of UPP1, alter lipid metabolism and stimulate production of eicosanoids, ceramide and reactive oxygen species that potentiate CNS injuries and certain neurological disorders [33]. Interestingly, this hypothesis offers an explanation for the multitude of beneficial effects of orally administered DHA and uridine on memory, neuronal health, regeneration and membrane synthesis in traumatic and chronic neuropathological conditions [33], [34].

The presented work demonstrates that γ-secretase is capable of influencing single gene transcription. However strong the impact will prove to be on the protein level of each single gene, we have further observed transcriptional effects spanning several genes throughout clearly defined pathways. This puts forth the possibility of much stronger effects on the target functions of these pathways than the small impact on the individual genes transcriptional or translational levels might indicate. In support of this hypothesis, we have observed that the proteins encoded by those genes interact with each other and are part of the Wnt pathways. Evaluation of the impact of these pathway-specific accumulative effects needs further investigation. This should include physiological and pathological *in vivo* experiments on both the transcript as well as protein levels. For γ-secretase to serve as a therapeutic target, it is indeed crucial to sharpen our view of its role and influence over gene transcription and biological functions.

## Materials and Methods

### Cell culture

The S-1 cell line overexpressing Flag-Pen2, Aph1-a2-HA, PS1 and NCT-GST [17], [79] was derived from the Chinese Hamster Ovary (CHO) γ-30 cell line [80] generated from the parental untransfected CHO cell line used in this study. All CHO cells were cultured in 10 cm dishes as biological triplicates in Dulbecco's modified Eagle's medium (DMEM) containing 10% Fetal Bovine Serum (FBS) and Penicillin/Streptomycin. The parental CHO cell line was treated with 10 µM of N-[(3,5-Difluorophenyl)acetyl]-L-alanyl-2-phenyl]glycine -1,1-dimethylethyl ester (DAPT) for 24 hrs. The S-1 cell line was supplemented with 200 µg/ml G418, 25 µg/ml puromycin, 250 µg/ml zeocin, 250 µg/ml hygromycin and 10 µg/mL blasticidin.

### RNA amplification and microarray analysis

CHO parental cell line triplicates were exposed for 24 hrs to the γ-secretase inhibitor DAPT (10 µM) in DMSO (0.05%), and S-1 cells were treated for the same time with DMSO (0.05%). Cells were next washed twice with PBS and total RNA was extracted, amplified, reversely-transcribed, labeled and hybridized to a 17 k mouse cDNA microarray chip produced by the DNA array facility of Lausanne (DAFL, see below).

*Total RNA extraction*: was performed using the RNeasy Mini Kit (Qiagen, Basel, Switzerland), in the absence of DNAse treatment. RNA quality was assessed using the RNA 6000 Nanochip assay (Agilent Technologies, Meno Park, USA) and RNA concentration was determined using the ND-1000 spectrophotometer (Nanodrop Technologies, Wilmington, USA). Three independent experiments were performed.

*RNA amplification*: a single round of amplification was performed with 3 µg of total RNA using the MessageAmp RNA Amplification Kit (Ambion, Austin, USA) and following the protocol provided with the kit. Next, 5 µg of amplified RNA was mixed with 9 µg random primers (Cat. No. 4819001; Invitrogen, Carlsbad, USA) in 19 µl of water, heated for 5 minutes at 70°C and then immediately transferred to ice.

*Reversed transcription and labeling*: was performed for 2 hrs at 42°C in a final reaction volume of 40 µl containing 1X SuperScript II buffer (Invitrogen), 40 units RNasin (Promega, Madison, USA), 10 mM DTT, 0.5 mM dATP, dGTP, dTTP, 0.2 mM dCTP, 0.1 mM of either Cy3-dCTP or Cy5-dCTP (GE Healthcare, Uppsala, Sweden) and 400 units of SuperScript II reverse transcriptase (Invitrogen). The RNA strand was hydrolyzed by adding 2 µl 500 mM EDTA and 4.5 µl 1 M NaOH and heating at 65°C for 15 minutes; the solution was then neutralized by adding 2.5 µl 1 M Tris (pH 6.8) and 4.5 µl 1 M HCl. The labeled cDNA was purified using the Qiagen MiniElute PCR Purification Kit (Cologne, Germany), eluting in 50 µl of EB buffer according to the manufacturer's instructions. The Cy3 and Cy5 labeled targets were combined and mixed with 400 µl of TE, 20 µg Cot 1 DNA (Invitrogen), 10 µg polyadenylic acid (Sigma, St. Louis, USA) and 10 µg yeast tRNA (Sigma). This mixture was concentrated to a final volume of 19.4 µl using a Microcon YM-30 filter (Millipore, Billerica, USA) according to the manufacturer's instructions. 20X SSC and 10% SDS were added to final concentrations of 3X and 0.4%, respectively, in a final volume of 24 µl. This mixture was heated for 2 minutes at 98°C, pipetted immediately onto the cDNA microarray and, after covering with a glass cover slip (Erie Scientific, Portsmouth, USA), placed in a humidified chamber (Telechem, Sunnyvale, USA) and allowed to hybridize at 64°C for 20 hrs. Slides were then washed at room temperature twice for 5 minutes in 2X SSC, 0.1% SDS, twice for 1 minute in 0.2X SSC, once for 1 minute in 0.1X SSC and once for 5 minutes in 0.1X SSC, 0.1% Triton X-100. After drying, slides were scanned on a microarray scanner (Agilent Technologies) and the resulting TIFF images were analyzed using the GenePix Pro 6.0 software (Molecular Devices, Sunnyvale, USA). The mouse cDNA microarrays used in this study consisted of approximately 17,000 PCR products generated from cDNA clones and control DNAs spotted onto Nexterion AL slides (Schott, Mainz, Germany). A complete description of the slides and their content can be obtained from the Lausanne DNA Array Facility (http://www.unil.ch/dafl). The microarray data set discussed in this publication has been deposited in the NCBI Gene Expression Omnibus (GEO, http://www.ncbi.nlm.nih.gov/geo/) and is accessible through GEO Series accession number GSE16379. Note that Hamster genomic sequence information is not yet sufficiently available to the research community. Consequently no commercial hamster-specific microarrays were available at the time of the experiment. However, the strategy to use a microarray from a closely related species is not new and has proven successful before [81].

### Statistical analysis of microarray results

The analysis was performed with open source R software packages (http://www.r-project.org/ and http://www.BioConductor.org/). Gene expression was quantified with the array package using print tip group lowess normalization without background subtraction. The resulting measures of expression for each array are the log2 ratios (M values) and the average log2 intensities (A value) of Cy3 and Cy5 signals. Statistics of differential expression between the different groups of samples were calculated with a linear model fitted by the limma package.

### RNA isolation for evaluation of microarray results

Total RNA was isolated with the RNeasy mini kit following the manufacturer's protocol for adherent cells in the case of CHO cell cultures. For the isolation of total RNA from brain tissue, the TRIzol reagent was used as described in the human samples section. RNA was dissolved in water, which was followed by ND-1000 spectrophotometer (Nanodrop Technologies, Wilmington, USA) quantification and pico chip quality control analysis (6000 Nanochip assay Agilent Technologies, Meno Park, USA).

### Reverse Transcription

Total RNA was reverse transcribed with our standard laboratory protocol. 1 µg of total RNA was dissolved in 4 µl of RNase-free water (Ultrapure DNase/RNase free water, Invitrogen Carlsbad, USA)) and premixed with 0.5 µg of oligo dT primer (synthesized by Eurogentec Seraing, Belgium) dissolved in 1 µl RNase-free water. The RNA/oligo dT premix was heated to 70°C for 5 minutes in a standard PCR machine (TProfessional Basic Gradient, Whatman Biometra Goettingen, Germany). The machine was paused to add 4 µl of 5X Buffer (ImProm-II M28A, Promega Madison USA), 4 µl of MgCl_2_ (25 mM) (Promega Madison USA), 1 µl dNTP Mix (10 mM U151B, Promega Madison USA), 1 µl RNase inhibitor (RNasin Plus N261A 40 u/ul Promega Madison USA), 1 µl of ImProm-II Reverse Transcriptase (Promega Madison, USA) and 4 µl RNase-free water. The PCR machine program was continued after pausing at 25°C for completion of reaction mixes with 60 min at 42°C and 15 min at 70°C. cDNA was kept at 4°C on wet ice for short-term or at −80°C for long-term storage.

### Real time PCR

Reverse transcription products were used without purification for real time PCR at equivalent of 0.5 ng/µl RNA in 384 well plates. Samples were used as biological triplicates and each one was additionally pipetted as a triplicate. Reaction volumes were 10 µl consisting of 5.02 µl SYBR Green (Power SYBR Green Master Mix #4367660 Applied Biosystems, Cheshire UK), 1.49 µl RT-PCR product at 0.5 ng/µl input RNA equivalent (0.75 ng/rxn) and 3.49 µl of 3 µM Forward and Reverse primer mix. 384 well plates were prepared with a liquid handling robot (Freedom EVOware Tecan Trading AG, Switzerland) and read for relative quantification with Applied Biosystems 7900HT Real-Time PCR System (Applied Biosystems, Cheshire UK). Primers (synthesized by Eurogentec Seraing, Belgium) for CHO cDNA were based on mouse code, which was aligned with rat and human code, preference was given to aligning sequences (Table 3). Sequence specificity was determined via nBlast. β-actin was used as housekeeping gene [82]–[89] for CHO as well as human cortex templates with the forward sequence: CCTTCAACACCCCAGCCATGTACG and the reverse sequence: CCTTCAACACCCCAGCCATGTACG.

**Table 3:** Sequences of primers used for real time PCR analysis with SYBR Green based on mouse code.

Gene	Forward	Reverse
UPP1	TCTACCATTTCAACCTCAGCACTAGCA	CCATGGCTCACAGACAGCACG
CLCN5	ATGGACTTCTTGGAGGAGCCA	GAGTTGAAGAGAATGGAATCAGGA
HES1	AAATGACTGTGAAGCACCTCCGG	GTCATGCAGTTGGCCAGGTGG
HHEX	CCGCTGTATGCGCCCACG	GCGTGCGTGTAGTCGTTCACC
SHMT1	TCTTGCTTAAATAACAAATACTCTGAGGG	GGCAGAGATTTTCTTCTTGTCTGTCAT
DVL3	GCTTCAATGGCCGGGTGG	CACTGCTCTGTTCTGTGGAGCTGC
WNT3A	CGAGGCCGGCGTTGGA	GACTGGCGATGGCCTGGC
CHRD	GGAGAGGGCTGCTATTTTGATGG	TCTGATTCTCTGGGAACCACTGCC
CDH15	CGCGTGCGGAGGGCC	GAAGCGATCAGTCTTCTCGCGG
MMP7	TAGGTGTGGAGTGCCAGATGTTGC	CATGACCTAGAGTGTTCCCTGGCC
GLP1R	ATAAGGACAACTCCAGCCTGCCC	TGCTGGGCAGCCGTGCTATAC
PNLIPRP	GCATCTGGGCGGGAACCC	TATCAGCATAGTGACCCATCTGTGGG
FGF20	CCTGCACGGCATCCTGCG	GTGCCCTGCACGCTGCC
PA2G4	CCAATAGAAGGTATGCTGTCACACCA	AAGGGACCACTGGTTATCCGCATGG
NTSR1	GACGGCGTTCACGCTGGC	AAGTTGTACAGCTCCACGGGCAT
SCT	CCCAGGGCCCGGCG	TCTGCGTCCTGCTCGCTGC
ITGBV	CCTGTGCCGGGAGGCAGA	GGCATTTGCATTCTCCACAGTGAC
IFT81	TGAGTTCAAGCGATACGTCA	GTTGATGCTGAATGGTTTCC
TERA	CAACCAGATCAGCAAACTGCAGAAG	AGCCATCTCTGTATCTGAGCCCTCA
PTPRG	TCTGGAGGATGATCTGGGAACAAAA	CACCCATGTCAGGCCACTGTGT
AMN1	GACAATGGTGTGGTTGCACTTGTTAGT	TGTTATCAGGGGGCAGCCATG

Primers were created for Tanneal of ∼58–59°C and nBlast was used to check for gene specificity.

### Statistical analysis of real time PCR results

Results were analyzed by the ΔΔCt method [90] and significance was calculated via students t-test. β-actin was used a normalizer to determine ΔCts. ΔΔCts were calculated against the mean of DAPT treated WT-CHO ΔCts or the mean of healthy human brain cortex ΔCts. Results were expressed as relative quantification by 2̂-(ΔΔCt) [90].

### Human samples

Human brain tissue was kindly provided by the Joseph and Kathleen Bryan Alzheimer's Disease Research Center, Duke University Medical Center. The Autopsy and Brain Donation procedures have been approved by the Duke University Institutional Review Board (IRB) and cortical brain tissue was obtained as described by [91]. 12 AD post-mortem confirmed cortical samples as well as 12 healthy cortical samples were obtained in dry ice. Cortical samples were of both genders, different ages, ApoE stati and Brack stages.

Isolation of total RNA: ∼50 ug of total cortex tissue were scraped off on dry ice three times for biological triplicates of each sample. TRIzol reagent (Invitrogen Carlsbad, USA) was used according to manufacturer's protocol for total RNA isolation. RNA was dissolved in water, which was followed by ND-1000 spectrophotometer (Nanodrop Technologies, Wilmington, USA) quantification and pico chip quality control analysis (6000 Nanochip assay Agilent Technologies, Meno Park, USA).

## Supporting Information

Dataset S1 EST clones with increased transcription under enhanced γ-scretase activity compared to inhibited γ-secretase activity. By applying a cut-off with a p value of 0.005 based on the false discovery rate (FDR, i.e. the probability to wrongly accept a difference between the two conditions), we found 2658 EST clones to be differentially expressed, with 1241 EST clones of increased with enhanced γ-secretase activity compared to inhibited γ-secretase activity. FC = Fold change; adj,P,Val = adjusted P-value
(0.20 MB XLS)

Dataset S2 EST clones with decreased transcription under enhanced γ-secretase compared to inhibited γ-secretase activity. By applying a cut-off with a p value of 0.005 based on the false discovery rate (FDR, i.e. the probability to wrongly accept a difference between the two conditions), we found 2658 EST clones to be differentially expressed, with 1417 EST clones of decreased transcription with enhanced γ-secretase activity compared to inhibited γ-secretase activity. FC = Fold change; adj,P,Val = adjusted P-value
(0.22 MB XLS)

Dataset S3 Molecular functional clusters of differentially transcribed genes as classified in the GO hierarchy. Lists of genes detected for differential transcription by the microarray, grouped in clusters of molecular function as defined by the GO hierarchy. Clusters are over- or underrepresented and do not indicate in- or decrease of the genes transcription levels.
(0.09 MB DOC)

Dataset S4 EST clones of transcriptional relevance differentially transcribed under enhanced γ-secretase compared to inhibited γ-secretase activity. By applying a cut-off with a p value of 0.005 based on the false discovery rate (FDR, i.e. the probability to wrongly accept a difference between the two conditions), we found 2658 EST clones to be differentially expressed with enhanced γ-secretase activity compared to inhibited γ-secretase activity. Among them 56 imply transcriptional relevance. FC = Fold change; adj,P,Val = adjusted P-value.
(0.03 MB XLS)
