# Supplementary material for: Gene Expression Profiling in Cells with Enhanced γ-Secretase Activity
Source: PLoS One. 2009 Sep 18;4(9):e6952. doi: 10.1371/journal.pone.0006952 (PMC2739295; doi:10.1371/journal.pone.0006952)
Supplement: Dataset S3 — Molecular functional clusters of differentially transcribed genes as classified in the GO hierarchy. Lists of genes detected for differential transcription by the microarray, grouped in clusters of molecular function as defined by the GO hierarchy. Clusters are over- or underrepresented and do not indicate in- or decrease of the genes transcription levels. (0.09 MB DOC) [file pone.0006952.s003.doc]

Over represented

[protein binding (GO:0005515)](http://amigo.geneontology.org/cgi-bin/amigo/go.cgi?view=assoc&search_constraint=terms&query=GO:0005515)

Slc12a6 Itm2b Cd8b1 Cops4 Sufu Arhgef12 Orc1l Glg1 Morf4l1 Klhl21 Foxp1 Plp1 Cdkn1a Timp1 Wwtr1 Bmp1 Ulk1 Ctnna2 Rassf1 Csh1 Gpbp1 Casp12 Kars Gtf2f2 Cd81 Nrp1 Ddx4 Hdac6 Capn2 Polb C80913 Mobkl1a Cdh16 Csf1 Setdb1 Cln6 Lrrc45 Col7a1 Ccr6 Pscd3 Pdlim5 Tyro3 Rad18 Atf4 Mki67ip Shc1 Anxa7 Centd2 Rab5a Ttf1 Syncrip Wnt7a Bclaf1 Gli2 AA673488 Cd47 Lima1 Chd8 Nip7 Cldn3 Cox4i1 Plaa Arl6 Sphk2 Tln1 Tax1bp1 Sgce Stat3 Tlr7 Atp5b Pcdh7 1200014M14Rik Pthlh Mlf1ip Id2 Limk2 Arih2 Dyrk1a Sec24c Tmpo Itga5 Ptpn13 Nid1 Lrpprc Cst3 Sst Rbpsuh Cct7 Unc84a Il12b Actn4 Gmfb Ntn1 Rybp Tpm2 Cluap1 Wnt9b Eln Cav3 Ywhah Rxra Psme1 Tcfap2c 6030408C04Rik Hoxb1 Map2k1 Gspt1 Mapk8 Adrb3 Rcbtb1 Actg1 Btg1 Itfg1 Fgf20 Vcl Ezh1 Fez2 Olfm1 Derl1 Rad21 Smarca2 Ccr2 Trps1 Ktn1 Eif3s1 Rgs12 Mef2d 4833420G17Rik Bfar Isgf3g Stk38 Sct Smg6 Gnpat Tiam2 Anxa11 Pcgf1 Arl2 Prim1 Bach1 Tcf2 Ptpn2 Porcn Klhl24 Ncl Lrrc8e Timp4 Tpt1 C3 Pafah1b1 Cul3 Sparc Itga1 Vdp Sip1 P4ha2 Crsp9 Ugcgl1 Numb Flna Atf7ip Fxyd5 Hmga2 Ptk2 Fgf14 Ctnnal1 Kpna3 Ergic3 Stx4a Hbp1 Gabarapl2 Dnajc3 Ela2 Ereg Tacc3 Rps2 Thrap3 Lrp2 Pfdn5 Arl8b D230025D16Rik Stk39 Mapk14 Dab2 2010204N08Rik Txndc4 Msln Rragc Snx10 Amotl2 Prlpl Nf2 Carhsp1 Ubtf Ccl19 Kctd2 Dcbld2 Cbx5 Igf2 Itgb5 Fyn Psenen 2900024C23Rik Src Axin1 Ccr10 3300001M20Rik Il1a Etf1 Itga8 Procr Alb1 1700081L11Rik Nap1l1 Cops3 Map3k3 Lmx1b Mllt4 Jarid1b Slc2a1 Jak2 Rad51 Myh10 Dab2ip Pcbp1 Lrpap1 Bnip1 Map2k4 Suv39h2 Ubox5 Aurka Aplp2 Prkcd Hspa5 Taldo1 Mmp14 Sdc2 Prpf31 Ephb2 Snrpa1 Epb4 Actr2 Cdh15 Mfge8 Cln5 Fgfr2 Zfpl1 1200015A19Rik Elf1 Dmd Cdc73 Trim37 Gsn Iqgap1 Eif3s4 Rai14 Mast2 Mrfap1 Mib1 Rchy1 Dvl3 Adam9 Lamc2 Grpel1 Eps8l2 Dstn Col6a1 Pigs Itga9 Ptch1 Arfrp1 Eif5a Trrap Ppp1r9b Eif2b1 Rere Rnf130 Ppp1ca Itgb1 Polr1e Dlk1 Hoxc8 Aph1a Inhbb Pex6 Ttc3 Ralbp1 Dlat Sec24b Bmp7 Exosc1 Il10ra AI450948 Phf17 Ptprn Sec23ip Ifrg15 Utrn Mxd4 Tm2d1 Cebpg Rac1 Errfi1 Rnf12 Bicd2 Aven Actn3 Phka2 Tpm1 Bub1b Atp2a2 Calm2 Ppil5 Skiv2l2 Ipo8 Ywhag Nr5a2 Pdzd3 Cd14 2810055G22Rik Ctsb Adam10 Bnip3l Cacybp Lsr Nedd4 Klhdc2 Casc3 Utp11l Arhgap21 Polr3f Pxdn Stmn1 Nrg4 Mbd1 Xrcc5 Eif2b2 Ppp3ca Jun Bche Adipor2 Kctd5 Dek Mtf2 Map4k5 Pdcd6 Atp5a1 Igf2r Dock7 Actb Cul7 Exosc8 Cbr2 Rab22a Spp1 Cd151 Tcf21 Ganab Pygo1 Thoc5 Eif2s1 Zc3hc1 Recc1 Paics Tsn Pxn Ruvbl1 Kif2c Spred1 Tlr2 Ctnnb1 Gtf2i Aes Jarid2 Alad AI428936 Trap1 Plod3 Arl6ip5 Smndc1 2310057M21Rik Cul2 Hsp90b1 Phf13 Myo5b Pole3 Nbl1 Ankib1 Hspa8 Hax1 Pmpca Phactr1 Crsp8 Unc5b Snx27 Nras Dynll1 Ccnb1 Fgf13 Pknox1 Kpna2 Ergic2 Pdrg1 Plec1 Hipk3 Elf4 Ccr8 Ube2a Farp2 Cdk4 Ptpre Orc4l Socs3 Il4 Ibrdc3 Hdac3 Mycbp Il18 H2-Ab1 Lmo7 Pxmp3 Rims2 Bhlhb8 Ltbp1 Ccl25 Ppia Lamb2 Gast Snx4 Mecr Dlx2 Kras Lama5 AU014645 D3Ertd300e Pfkm Trp53 Ldha Plk1 Il1rn Ccnd2 Ncf1 Rad1 Rab7l1 Fn1 Notch3 Itga7 Fkbp10 Casp9 Hspd1 H47 Nsmaf Ube4b Camk2g Canx Dtprp Icosl Acvr1b Jarid1a S100a6 Nisch Bcap31 Tpm4 Cntn4 Timeless Kif1b Gas1 Oxsr1 Nfya Nfx1 Fga Suv39h1 Apoa1 Casp4 Lrp8 Eps15 Ihh Sdc1 Tmem33 S100a1 Drg1 Plekhc1 Dync1h1 Rbl2 Smarca4 Fgfr1 Prkcz Eif4a1 Llgl1 Plxnb2 Atf2 Trib3 Ddx56 Anxa5 Hgf Chrd Lamc1 Ahsa1 Mtch1 Marcks Nxf1 Magoh Efemp2 Ttn Creb1 Pbx1 Dcn Cd9 Cldn1 Ccne1 Gars Prkcbp1 Inhba Alcam Cul5 Ptprz1 Egfr Itga3 Ptpn11 Mtmr2 Asxl1 Gatad2b Tollip Mxd3 Cct5 Rnf168 Fgf8 Add1 Iapp Rrm2 Dkk1 Shcbp1 Actn2 Tnc Actr8 Fancl Spna2 Myh3 Calm1 Cav1 Abcd2 Dnajc5 Sh3kbp1 Zfp110 Synj2bp Bzw2 Cdk7 D6Wsu176e Fbxl10 Mxi1 Cdc20 Robo1 Tcte3 Vtn Ccl5 Fth1 Sipa1l1 Rb1 Fgf11 Ap1g1 Psmc5 Mtap4 Phf3 Pgrmc2 Dll4 Tdgf1 Coro1b Tnfrsf19 Dbf4 Tef Pros1 Homer2 Snapap Glcci1 Mtf1 Wnt6 Rpp14 Gadd45b Hspe1 Cxxc1 Neurog2 Trim3 Ppp2ca Arpc1a Sfrs7 Tcf20 Insr Lmtk2 Cd34 Rhoa Cdh5 Ecd Pld1 Neo1 Foxp2 Tpd52l2 Il8rb Fasn Cd40lg Rest Cdkn1b Timp2 Bysl Dsg3 Mcl1 App Bnip3 Ptrf Smarcc1 Mycl1 Cd82 Tcp1 Sdc4 Surb7 Ncor2 Ap2a1 Txnip Atrnl1 Appbp2 Ddx20 Fgf12 Gins4 Senp3 Hif1a Hspg2 Nsf Strn4 Atoh1 Coro1c Scmh1 Farp1 Gmnn Pa2g4 Wt1 Hexa Abi2 Vegfc Myst4 Rims1 Tmsb4x Zfp36l1 Parva Ccl24 Gdf5 Hoxa13 Smurf1 Grb2 Ebna1bp2 Ddx39 Pcsk2 Cldn4 Pdlim1 Ash2l Tbp Acy3 Fmn2 Ppy Jmjd2b Tmem11 Rnf34 Ppp1cc Bub3 Ik Arpp19 Med6 Fzd5 Ccnd1 Sec24d Tep1 2610033H07Rik Rps27 Eif2ak1 Sfrs3 Nid2 Ctr9 Stk25 Ern1 Spnb2 Cdh1 Kntc2 Rab10 Csnk2a2 Ybx1 Taf3 Furin Vim Cxcl12 Mll3 Lamb1-1 Tgoln1 Rcbtb2 Xab2 Tnnt1 Fcgrt Strap Pcna B2m Trip13 Phr1 Ppp1r13b Eif2b4 Ccr3 Jup Gata3 Calr Igfbp4 Vldlr Bmpr1b Col1a2 Dock9 Cdon Cd44 Dnaja3 Stk3 Grem2 March1 Ube2d3 Cetn2 Dach1 Prkra Tac1 Wnt10a Vcam1 Styx Rab3d Cdkn2b Cdh8 Cog4 Nup153 LOC434860 Ttc1 Lhx9 Sema3c Dnajc11 Fzd1 Dysf Acvrl1 Tmod2 Muc1 Itga2 6330505N24Rik Hoxb9 Txnl2 Cct4 Rnf167 Clstn1 Kpnb1 Was Actn1 Ncoa1 Rabl3 Bcar1 Trip4 Flnb Ddef1 Shh Abtb2 Tbc1d8 Tle1 Prrx1 Adm Sin3a Cdk6 Sertad2 Exoc4 Rtn3 Hdgf Met Apex1 Nrip1 Pcolce Rab6ip1 Psen1 Hdac5 Nr2c2 Il17ra Atp1a1 Mlph F2r Map1lc3b Ihpk1 Rnf44 Jag2 Polr2g Coro1a Tmed9 Ptgfrn Dag1 Myo1e Tceb3 Ptprs Rala Ctsl

[nucleotide binding (GO:0000166)](http://amigo.geneontology.org/cgi-bin/amigo/go.cgi?view=assoc&search_constraint=terms&query=GO:0000166)

Pck2 Tarsl1 Nxf1 Ttn Slc30a9 Gars Rps24 Egfr Adss Cct5 Ddx31 Myh3 Ppil4 Abcd2 Cdk7 Psmc5 Ddx52 Hspe1 Aars Ptov1 Csnk1d Sfrs7 Oas1c Insr Sfrs10 Lmtk2 Itm2c Tnrc6a Rhoa Neo1 Eef1a1 Supv3l1 Ddx18 Ak3l1 Dhx8 Tcp1 Tubb2c Ddx20 Gtpbp4 Fgfr4 Nsf Psmc1 1300010F03Rik Ddx39 Nt5c Pfkl 2310022K01Rik Mov10 Tep1 Ddx41 Eif2ak1 Sfrs3 Pank3 Cpeb2 Stk25 Ern1 Rabl2a Rab10 Csnk2a2 Wars Hbs1l Trip13 Mtg1 Ddx55 4921513D23Rik Bmpr1b Rbmx Fignl1 Dock9 Mtif2 Stk3 Cetn2 Rab3d Chdh Rab24 Acvrl1 Arf3 Cct4 Cirbp Rabl3 Ak2 Cdk6 Gss Met Riok2 Nr2c2 Atp1a1 Tubb3 Atad3a Arf4 Myo1e Atp11a Gnai1 Rala Ndufv1 Itm2b Tgfbr2 Rab20 Orc1l C230081A13Rik Rab13 Ulk1 Glud1 Kars Gtf2f2 Dhx29 Ddx4 Igf2bp1 Cdh16 Setdb1 Pcsk6 Acox1 Tyro3 Mki67ip Rab5a Syncrip Pabpc1 Txnrd1 Tubg2 Chd8 Eif5b Arl6 Sphk2 Atp6v1a Atp5b Rab14 Limk2 Myo1a Dyrk1a Uck2 Pank2 Stk24 Cct7 Abce1 Map2k1 Lamp2 Gspt1 Mapk8 Actg1 Sbk1 Rbm18 Smarca2 Epha2 Itpk1 Iars Mcm5 Pink1 Bmpr1a Prkar2b Dld Stk38 Pygl Hspa14 Ppox Arl2 Ncl Timp4 Farslb Acads Pskh1 Ephb6 Ptk2 Gabarapl2 Gsr Thrap3 Abcc3 Riok1 Tbrg4 Arl8b Pabpc4 Stk39 Mapk14 Abcb6 Psmc3 Pdk1 Kifc3 Rragc Trnt1 D330037H05Rik Fyn Src Sars2 Centg3 Map3k3 Opa1 Jak2 Rad51 Myh10 Kif19a Map2k4 Gtf2f1 Aurka Prkcd Hspa5 Rbm28 Ephb2 Epb4 Drg2 Gtpbp2 Fgfr2 Pcsk5 Tek Eif3s4 Mast2 Grpel1 Rarsl Arfrp1 Eif2b1 Cabc1 Srpr Pgk1 Bub1 Pex6 E130016E03Rik Atp8b1 Hnrpdl 2610019A05Rik Rars Cugbp2 Rac1 Dscr1l2 Bub1b Atp2a2 Skiv2l2 Pnpo Mbd1 Xrcc5 Eif2b2 Mat2a Dek Map4k5 Atp5a1 Dock7 Actb Rab22a Recc1 Eif5 Paics Ruvbl1 Kif2c Tgm2 Sars Trap1 Arl5c Axl Farsla Top2a 2310057M21Rik Trio Hsp90b1 Myo5b Hspa8 Rbm3 Ckmt1 Igf2bp3 Nras Gfm2 Bcs1l Hipk3 Guf1 Cdk4 Nek8 Orc4l Ccrk Gbp2 Dcakd U2af1l4 Speg Nav3 Kifc2 Kif27 Ankk1 Kras Pfkm Plk1 Rab7l1 Mod1 Hspd1 Prkcn Camk2g 0610009D07Rik Acvr1b Inoc1 Cntn4 Kif1b Oxsr1 Ankrd17 Hspa4 Melk Pdgfrb Drg1 Cdc42bpa Dync1h1 Smarca4 Fgfr1 Epha4 Prkcz Eif4a1 Trib3 Ddx56

[transferase activity (GO:0016740)](http://amigo.geneontology.org/cgi-bin/amigo/go.cgi?view=assoc&search_constraint=terms&query=GO:0016740)

Map4k5 Trmt5 Zc3hc1 Sdf2 Tgm2 Axl Trio Pole3 Ckmt1 Ccnb1 Mettl2 Plec1 Hipk3 Cdk4 Nek8 Cpt1a Ccrk Gnpnat1 Nmt1 Dcakd Zdhhc20 Speg Gsta1 Ankk1 Aup1 Pfkm Plk1 Prkcn Camk2g Zdhhc5 Acvr1b Hs3st1 Tpst1 Mgat1 Asnsd1 Oxsr1 Suv39h1 Stt3a Ihh Melk Pdgfrb Cdc42bpa Dph5 Fgfr1 Epha4 Prkcz Trib3 Pck2 Ttn Fdft1 Elovl6 Cdkn2c Ccne1 Prkcbp1 Tfb1m Rg9mtd1 Egfr Ndst2 Tnks2 Gpt2 Ppat Cds2 Sh3kbp1 Cdk7 Pbef1 Mett10d Sult2a2 Ddost Psmc5 Gstm5 Qtrtd1 Dbf4 Oat Dgkg Ate1 Csnk1d Oas1c Insr Lmtk2 Rnmtl1 4930402E16Rik Neo1 Mettl6 Tusc3 Fasn Cdkn1b Dgkb Pole2 Ak3l1 Surb7 Hif1a Fgfr4 Myst4 Pcsk2 Soat1 Pfkl B3gnt5 Aytl2 Ccnd1 Tep1 Icmt Eif2ak1 Pank3 Stk25 Ern1 Hmgcs2 Pnpt1 Tkt Csnk2a2 Mll3 Srm Strap Pcna Twistnb Shmt1 Bmpr1b Pkm2 Pigq Stk3 Csrp2bp Cdkn2b Acvrl1 BC023882 Plau Ncoa1 Chst11 Ak2 Got2 Cdk6 Met Riok2 Nr2c2 Upp1 Ihpk1 Gsta3 Polr2g Papd1 Cox10 Bcat2 Oxct1 Tgfbr2 Xylt2 Mettl5 Cdkn1a C230081A13Rik Ulk1 Ccnh Nrp1 Polb Glt8d1 Mobkl1a Setdb1 Tyro3 Mettl7a Fbl Sphk2 Mlf1ip Limk2 Dyrk1a Uck2 Pank2 Stk24 Hmgcs1 Parp16 Map2k1 Casd1 Mapk8 Ggps1 Sbk1 Epha2 Itpk1 Pink1 Bmpr1a Prkar2b Stk38 Pygl Gnpat Adpgk Prim1 Large Porcn Extl3 Pskh1 Ugcgl1 Ephb6 Ptk2 B4galt4 Nadk Riok1 Tbrg4 Stk39 Mapk14 Polr3c Pdk1 Trnt1 Pip5k2c Fyn 2900024C23Rik Bcat1 Src Map3k3 Dlst Jak2 Mgat2 Mettl4 Brd2 Map2k4 Suv39h2 Ubox5 Stt3b Gtf2f1 Aurka Prkcd Taldo1 Ephb2 Fgfr2 Gbe1 Tek Mast2 Pet112l Trrap Ext1 BC068171 Cabc1 Pgk1 Bub1 Polr1e Dlat Pofut1 2610019A05Rik Gpam Phka2 Bub1b D5Wsu178e 4930548G07Rik Usp14 Polr3f Wdr62 Polr2i Mat2a

[transcriptional activator activity (GO:0016563)](http://amigo.geneontology.org/cgi-bin/amigo/go.cgi?view=assoc&search_constraint=terms&query=GO:0016563)

Ctnnb1 Crsp8 Elf4 Usf2 Nfya Atf2 Ccne1 Cdk7 Rb1 Tef Mtf1 Cxxc1 Tcf20 Ecd Smarcc1 Myst4 Pdlim1 Gata3 Hoxb9 Ncoa1 Trip4 Prrx1 Apex1 Nrip1 Wwtr1 Atf4 Gli2 Stat3 Rbpsuh Etv5 Ywhah Rxra Mef2d Tcf2 Crsp9 Thrap3 Foxc2 Sertad2 Gtf2f1 Elf1 Trrap Onecut1

[nucleic acid binding (GO:0003676)](http://amigo.geneontology.org/cgi-bin/amigo/go.cgi?view=assoc&search_constraint=terms&query=GO:0003676)

Nol5 Satb1 Hbs1l Pcna Eif2b4 Gata3 Calr Eif3s2 Ddx55 4921513D23Rik Rbmx Nkd2 Mtif2 Cetn2 Dach1 Prkra Cdkn2b Zfp281 Tcf3 Hoxd13 Lhx9 Banf1 BC023882 Hoxb9 Lrrfip1 Hes3 Cirbp Ncoa1 Rabl3 Ddef1 Prrx1 Apex1 Pcolce Riok2 Nr2c2 Hoxa1 Irx3 Foxa2 Polr2g Mrpl3 Papd1 Snapc1 Neurog1 Tceb3 Hmgn2 Secisbp2 Arhgef12 Orc1l Ankrd26 Hdlbp Foxp1 Mettl5 Neurod4 Gpbp1 Kars Gtf2f2 Dhx29 Ddx4 Polb Igf2bp1 Eif1a 5730601F06Rik Setdb1 Pcsk6 Rpl7 Eomes Rad18 Atf4 Sfrp4 Mki67ip Centd2 Ttf1 Syncrip Pabpc1 Bclaf1 Gli2 Chd8 Nip7 Eif5b Fbl Rnaset2 Zfp687 Pogk Tax1bp1 Stat3 Tlr7 Thap11 Arih2 Tmpo Lrpprc Rbpsuh Unc84a Etv5 Hoxd1 Rybp Rabgap1 Zc3h13 Rxra Tcfap2c Hoxb1 Gspt1 Foxb2 Ezh1 Rbm18 Rad21 Cited1 Smarca2 Nucb2 Zfp354a Trps1 Eif3s1 Mcm5 Mef2d Isgf3g BC057593 Zcchc7 Phf5a Smg6 Eif2s2 Bach1 Tcf2 Ncl Lhx8 Sip1 Hes2 Eif1 Hmga2 Endog Hbp1 2310002B06Rik Rps2 Pabpc4 Eif4g1 Rpa1 Polr3c Csdc2 Foxa1 Zfp219 Carhsp1 Trnt1 Ubtf D330037H05Rik Stat6 Bcorl1 3300001M20Rik Etf1 Alb1 Centg3 Lmx1b Ddb1 Gtf2h1 Jarid1b Ankrd25 Mettl4 Rad51 4930432O21Rik Ccar1 Pcbp1 Mrpl21 Foxc2 Hoxb4 Gtf2f1 Aplp2 Rbm28 Asb3 Snrpa1 D19Bwg1357e Gtpbp2 Zfpl1 Elf1 Dlx4 Sf3a3 Eif3s4 Rai14 Zfp143 Mbnl3 Tcf25 Eif5a Pet112l Usp39 Eif2b1 Rere Lgtn BC068171 Srpr Polr1e Nfib Hoxc8 E130016E03Rik Exosc1 Hnrpdl Mxd4 Cebpg Cugbp2 Hes5 Onecut1 Dscr1l2 Skiv2l2 4930548G07Rik Nr5a2 Zfp422 Lsr Gpatc4 Polr3f Irx5 Mbd1 Egr1 Endod1 Nucb1 Xrcc5 Auh Eif2b2 Jun Polr2i Dek Mtf2 Ets2 Tshz1 Exosc8 Tcf21 Tgif2 Nfkbiz Eif2s1 Recc1 Eif5 Tsn Kif2c Gtf2h4 Ctnnb1 Gtf2i Jarid2 Tsc22d1 Zfp622 Smndc1 Top2a Pole3 Ankib1 Hes1 Gtf3a Rbm3 Igf2bp3 Gfm2 Pknox1 Bicc1 Set Fubp1 Hhex Cebpz Elf4 Hp1bp3 Guf1 Orc4l Eif3s7 Zbtb25 Hdac3 U2af1l4 Usf2 Gtf3c1 Bhlhb8 2610507B11Rik Dlx2 Lama5 AU014645 Trp53 Rad1 Exosc4 Notch3 Zfp213 0610009D07Rik Zfp710 Inoc1 Jarid1a Timeless Ankrd17 Nfya Nkrf Nfx1 2010003J03Rik Rbl2 Smarca4 Eif4a1 Evx1 Atf2 Zfp62 Ddx56 Cxxc5 Zcchc9 Nxf1 Magoh Creb1 Pbx1 Iqwd1 Cdkn2c 2410018L13Rik Suhw4 Mtdh Nfia Tsc22d4 Rps24 Tfb1m Rg9mtd1 Egfr Gatad2b Txndc14 Mxd3 Ddx31 Tulp4 Ppil4 Cggbp1 Zfp110 Bzw2 Fbxl10 Mxi1 Nol5a Rb1 Cpsf3 Phf3 Peg10 C330002I19Rik Wbp7 Dbf4 Tef Ddx52 Mtf1 Rpp14 Cxxc1 Neurog2 Aars Zbtb10 Sfrs7 Tcf20 Oas1c Sfrs10 Rnmtl1 Ccdc16 Tnrc6a Tieg3 Foxp2 D8Ertd457e Lhx6 Hnf4a Rest Eef1a1 Supv3l1 Zfp706 Ddx18 App Hoxb6 Pole2 Ptrf Smarcc1 Dhx8 Mycl1 Foxn2 Ncor2 Psip1 Ddx20 Hif1a Cdx1 Rpl32 Atoh1 Pa2g4 Wt1 Tdrd7 Myst4 D630045J12Rik Zfp36l1 Prpf4 BC003993 Hoxa13 Ddx39 Ash2l Tbp Jmjd2b Rnf34 2310022K01Rik Tep1 Ddx41 2610033H07Rik Rps27 Eef1d Eif2ak1 Sfrs3 Cpeb2 Ern1 Spnb2 Slc6a8 Pnpt1 Ybx1 Emg1 Mll3 Dnase2a

[isomerase activity (GO:0016853)](http://amigo.geneontology.org/cgi-bin/amigo/go.cgi?view=assoc&search_constraint=terms&query=GO:0016853)

Top2a Srr Ppia Fkbp10 Trub1 Pdia3 Ppil4 Ppid Mpi1 Hnf4a Txndc10 Cpd Pdia6 Ppig Ptges Idi1 Ppil3 Nr2c2 Txndc5 Fkbp9 Rpe Tpi1 Pdia5 Rxra Itpk1 Txndc4 BC068171 Pdia4 Ppil5 Pgm2

[hydrolase activity (GO:0016787)](http://amigo.geneontology.org/cgi-bin/amigo/go.cgi?view=assoc&search_constraint=terms&query=GO:0016787)

Atp5a1 Cfi Amy2 Exosc8 Rab22a Ganab Afp Recc1 Ruvbl1 Tgm2 Plcg2 2310057M21Rik Man2b1 Acp5 Ctsc Hspa8 Mmp17 Pmpca Nras Gfm2 Bcs1l Guf1 Ptpre Ela1 Orc4l Gbp2 Hdac3 Hrsp12 Nav3 Lamb2 Kras Glb1 Smpd1 Rad1 Rab7l1 Exosc4 Casp9 Nup160 Spint2 Lgmn Camk2g Arhgef10 Inoc1 6530401N04Rik Lpl Cntn4 Kif1b Asnsd1 Acp1 Isg20l1 Atp1b3 Casp4 Mmp13 Ppm1m Ihh Dync1h1 Smarca4 Osgepl1 Eif4a1 Ptpra Ddx56 Ptrh2 Hgf Ttn Usp38 Prss3 Cdc14b Npepps Atp6v0b Ptprz1 Ptpn11 Mtmr2 Ndst2 Ddx31 Dpysl2 H6pd Senp7 Abcd2 Ptplb Pitrm1 Cpsf3 Psmc5 Plcd3 Trfr2 Ddx52 Rpp14 Ppp1r3b Btd Ppp2ca Man1a Rhoa Pld1 Fasn Eef1a1 Ddx18 Dhx8 C1s Tubb2c Ddx20 Cpd Senp3 Nln Nsf Pa2g4 Pepd Pop1 Hexa Pnliprp1 D6Wsu116e Psmc1 1300010F03Rik Ddx39 Pcsk2 Clpp Nt5c Acy3 Ppp1cc Mov10 Ddx41 Adam17 Ern1 Pnpt1 Impad1 Furin Usp16 Dnase2a Acpl2 Atp5o Hbs1l Trip13 Uchl5 Ddx55 Fignl1 Mtif2 Cetn2 Ptprcap Ada Styx Rab3d Sulf2 Atp5f1 Idi1 Plau Tatdn1 Rnf167 Shh Ptprg Alkbh3 Apex1 Psen1 Hdac5 Atp1a1 Hyal2 Tubb3 Jag2 Atad3a Atp11a Gnai1 Ptprs Rala Ctsl Orc1l Rab13 Bmp1 Casp12 Gtf2f2 Dhx29 Ddx4 Hdac6 Capn2 C1r Pcsk6 Ptp4a1 Ppm1d Rab5a Apobec3 E330036I19Rik Tubg2 Eif5b Rnaset2 Pcsk1 Psmb3 Hexdc Fuca1 Atp6v1a Atp5b Rab14 Pappa Ptpn13 Abce1 Tasp1 Usp15 Dirc2 Atp1b1 Gspt1 Mapk8 Mmp11 Tfrc Itpk1 Apeh Mcm5 Prcp Hspa14 D19Wsu12e Ptpn2 Tbl3 Pafah1b1 Ctsd Atf7ip Endog Ptprf Ela2 Abcc3 Arl8b Abcb6 Psmc3 Rragc Plcd1 Mrpl46 Pcsk4 Naglu Usp4 Hdhd3 Plcb1 Gm2a Adam19 Usp32 Opa1 Rad51 Myh10 Gaa 2010111I01Rik Abhd4 Mmp14 Eps8 Gtpbp2 Pcsk5 Gbe1 Man1a2 Adam9 Pigs Arfrp1 Usp39 Lap3 Srpr Rnf130 Ppp1ca Aph1a Hars2 Pex6 E130016E03Rik Mbtps1 Exosc1 Atp8b1 Ptprn Ddhd1 Rac1 Qrsl1 Atp2a2 Skiv2l2 Ipo8 Aga Usp14 Ctsb Adam10 Endod1 Xrcc5 Ppp3ca Bche Cdc25a

[ion binding (GO:0043167)](http://amigo.geneontology.org/cgi-bin/amigo/go.cgi?view=assoc&search_constraint=terms&query=GO:0043167)

1600014K23Rik Rpa1 Acsl1 Rragc Plcd1 Zfp219 Trnt1 Stat6 Slc31a2 Plcb1 Alb1 Adam19 Centg3 Usp32 Map3k3 Lmx1b Opa1 Jarid1b Limd2 4930432O21Rik Ccar1 Lrpap1 2010111I01Rik Suv39h2 Setd2 Ubox5 Gtf2f1 Prkcd Hspa5 Mmp14 Lias Eps8 Hrbl Cdh15 Cycs Slc15a4 Cspg2 Gbe1 Zfpl1 Dmd Man1a2 Trim37 Gsn Sf3a3 Mast2 Sc4mol Mib1 Rchy1 Adam9 Zmynd12 Zfp143 Ptch1 Ftl1 Mbnl3 Sepx1 Usp39 Trrap Lap3 Rere Rnf130 Ppp1ca Dlk1 Ttc3 Armc1 Mbtps1 Sec24b Pofut1 Phf17 Sec23ip Utrn Asph Pdia4 Ddhd1 Rnf12 Actn3 Atp2a2 Eml2 Calm2 Slc11a2 Nr5a2 2810055G22Rik Adam10 Zfp422 Cyp46a1 2310047O13Rik Pgm2 Pxdn Mbd1 Egr1 Nucb1 Rasa3 Ppp3ca Polr2i Mat2a Mtf2 Pdcd6 Cyc1 Atp5a1 Tshz1 Amy2 Hba-a1 Pygo1 Afp Cyb5b Zc3hc1 Recc1 Pxn Tgm2 Nbr1 Alad Egln1 Zfp622 Plod3 Man2b1 Hsp90b1 Phf13 Acp5 Ctsc Ccdc65 Ankib1 Gtf3a Mmp17 Lyar Elf4 Myl6 Nek8 Ndufs1 Lrp1 Ibrdc3 Zbtb25 Lmo7 Pxmp3 U2af1l4 Rims2 Zdhhc20 Ltbp1 Eno1 Lamb2 2610507B11Rik Mecr Spire1 Pfkm Glb1 Trp53 Ssr1 Notch3 Itga7 Fkbp10 Mod1 Prkcn Zfp213 Zdhhc5 Canx Acvr1b Zfp710 Jarid1a S100a6 Oxsr1 Nfx1 Adcy7 Fhl1 Atp1b3 Suv39h1 Lrp8 Eps15 Mmp13 Ppm1m S100a1 Osgepl1 Prkcz Atf2 Zfp62 Anxa5 Pck2 Cxxc5 Zcchc9 Sdhb Efemp2 Fdft1 Fstl1 Rcn2 Suhw4 Prkcbp1 Npepps Atp6v0b Ptprz1 Smap1l Egfr Asxl1 Gatad2b Adss Rnf168 Ddx31 Add1 Rrm2 Actn2 Fancl Spna2 Calm1 Zfp110 Fbxl10 Fth1 Nsmce1 Pitrm1 Phf3 Leprel2 Hbb-b1 Peg10 Plcd3 Pgrmc2 Dll4 Cab39 Wbp7 Dbf4 Pros1 Mtf1 Dgkg Cxxc1 Trim3 Ppp2ca Zbtb10 Sfrs7 Tcf20 Slc12a7 Man1a Ccdc16 Mpi1 Tieg3 Cdh5 Foxp2 D8Ertd457e Lhx6 Hnf4a Rest Dgkb Zfp706 Dsg3 App C1s Cpd Nln Nsf Pa2g4 Pepd Wt1 Hexa D6Wsu116e Myst4 Rims1 Zfp36l1 Map3k7ip2 Pdlim1 Ash2l Nt5c Mvp Jmjd2b Pfkl Rnf34 Ppp1cc Cyp17a1 Mbtd1 Pcdhgc3 Aytl2 Sec24d Ddx41 Rps27 Clcn5 Nid2 BC003331 Adam17 Stk25 Rgnef S100a13 Ern1 Slc6a8 Cdh1 Tkt Taf3 Furin Usp16 Mll3 Atp5o Phr1 Fgd6 Gata3 Calr Vldlr Bmpr1b Oaz2 Nkd2 Pkm2 Dnaja3 Stk3 March1 Cetn2 Sdf4 Ada Sulf2 Zfp281 Cdh8 Nup153 Idi1 Lhx9 Acvrl1 Jmjd1a Itga2 Rnf167 Clstn1 Kpnb1 Actn1 Trip4 Ddef1 Ptprg Scnm1 Apex1 Slc38a4 Nr2c2 Atp1a1 Mlph 1700052N19Rik Leprel1 Rnf44 Jag2 Fkbp9 Cyp7a1 Dag1 Atp11a Slc12a6 Ndufv1 Tgfbr2 Ptgs2 Arhgef12 Chordc1 Foxp1 Cdkn1a Bmp1 Rassf1 Nmd3 Adcy9 Hdac6 Capn2 Polb C1r Mobkl1a Cdh16 Scd1 5730601F06Rik Setdb1 Pcsk6 Pdlim5 Rad18 Anxa7 Ppm1d Centd2 Gli2 AA673488 Apobec3 Lima1 E330036I19Rik Pcsk1 Zfp687 Hexdc Fuca1 Tax1bp1 Atp6v1a Sgce Stat3 Atp5b Thap11 Pcdh7 Pthlh Limk2 Arih2 Pappa Sec24c Itga5 Nid1 Unc84a Actn4 Abce1 Rybp Zc3h13 Rxra 6030408C04Rik Atp1b1 Kcnab1 Mmp11 Hbld2 Rad21 Acsl5 Itpk1 Nucb2 Zfp354a Trps1 Eif3s1 Pink1 Bmpr1a Nkd1 Bfar BC057593 Stk38 Zcchc7 Adpgk Anxa11 D19Wsu12e Eif2s2 Pcgf1 Prim1 Lhx8 Tpt1 Extl3 Sparc Itga1 P4ha2 Lman2 Endog B4galt4 Lrp2 Nadk

[transcriptional repressor activity (GO:0016564)](http://amigo.geneontology.org/cgi-bin/amigo/go.cgi?view=assoc&search_constraint=terms&query=GO:0016564)

Aes Jarid2 Hes1 Hipk3 Nkrf Trib3 Mxi1 Rb1 Foxp2 Rest Ncor2 Ddx20 Scmh1 Myst4 LOC434860 Tle1 Sin3a Apex1 Nrip1 Hdac5 Sufu Foxp1 Wwtr1 Fabp4 Bclaf1 Id2 Rybp Trps1 Atf7ip Pfdn5 Mxd4 Rnf12 Mbd1

[ligase activity (GO:0016874)](http://amigo.geneontology.org/cgi-bin/amigo/go.cgi?view=assoc&search_constraint=terms&query=GO:0016874)

Paics Sars Farsla Ube2a Smurf2 Ube4b Rwdd4a Asnsd1 Tarsl1 Gars Adss Fancl Fbxo21 Aars Ptov1 Ube2r2 Smurf1 Rnf34 Wars Phr1 Ube2d3 Rnf167 Ube2g1 Gss Ube2t Ube2o Kars Fbxo3 Ctps2 Cdh16 Anapc4 6030408C04Rik Lamp2 3110006E14Rik Acsl5 Iars Farslb Acsl1 Sars2 Ubox5 Mib1 Ube2e2 Rarsl Pet112l Hars2 Ttll6 Rars Rnf12 Ube3b 2810055G22Rik Nedd4

[kinase regulator activity (GO:0019207)](http://amigo.geneontology.org/cgi-bin/amigo/go.cgi?view=assoc&search_constraint=terms&query=GO:0019207)

Cdkn1b Ccni Ccnd1 Cdkn2b Bccip Cdkn1a Mobkl1a Prkar2b Dnajc3 Ywhag Ccnb1 Fgf13 Socs3 Ccnd2 Trib3 Cdkn2c Ccne1

[purine nucleotide binding (GO:0017076)](http://amigo.geneontology.org/cgi-bin/amigo/go.cgi?view=assoc&search_constraint=terms&query=GO:0017076)

Gene list:Nras Gfm2 Bcs1l Hipk3 Guf1 Cdk4 Nek8 Orc4l Ccrk Gbp2 Dcakd Speg Nav3 Kifc2 Kif27 Ankk1 Kras Pfkm Plk1 Rab7l1 Mod1 Hspd1 Prkcn Camk2g Acvr1b Inoc1 Cntn4 Kif1b Oxsr1 Ankrd17 Hspa4 Melk Pdgfrb Drg1 Cdc42bpa Dync1h1 Smarca4 Fgfr1 Epha4 Prkcz Eif4a1 Trib3 Ddx56 Pck2 Tarsl1 Ttn Gars Egfr Adss Cct5 Ddx31 Myh3 Abcd2 Cdk7 Psmc5 Ddx52 Hspe1 Aars Ptov1 Csnk1d Oas1c Insr Lmtk2 Itm2c Rhoa Neo1 Eef1a1 Supv3l1 Ddx18 Ak3l1 Dhx8 Tcp1 Tubb2c Ddx20 Gtpbp4 Fgfr4 Nsf Psmc1 1300010F03Rik Ddx39 Pfkl Mov10 Tep1 Ddx41 Eif2ak1 Pank3 Stk25 Ern1 Rabl2a Rab10 Csnk2a2 Wars Hbs1l Trip13 Mtg1 Ddx55 Bmpr1b Fignl1 Dock9 Mtif2 Stk3 Cetn2 Rab3d Chdh Rab24 Acvrl1 Arf3 Cct4 Rabl3 Ak2 Cdk6 Gss Met Riok2 Nr2c2 Atp1a1 Tubb3 Atad3a Arf4 Myo1e Atp11a Gnai1 Rala Itm2b Tgfbr2 Rab20 Orc1l C230081A13Rik Rab13 Ulk1 Glud1 Kars Gtf2f2 Dhx29 Ddx4 Cdh16 Pcsk6 Acox1 Tyro3 Rab5a Txnrd1 Tubg2 Chd8 Eif5b Arl6 Sphk2 Atp6v1a Atp5b Rab14 Limk2 Myo1a Dyrk1a Uck2 Pank2 Stk24 Cct7 Abce1 Map2k1 Lamp2 Gspt1 Mapk8 Actg1 Sbk1 Smarca2 Epha2 Itpk1 Iars Mcm5 Pink1 Bmpr1a Prkar2b Dld Stk38 Hspa14 Ppox Arl2 Timp4 Farslb Acads Pskh1 Ephb6 Ptk2 Gabarapl2 Gsr Thrap3 Abcc3 Riok1 Tbrg4 Arl8b Stk39 Mapk14 Abcb6 Psmc3 Pdk1 Kifc3 Rragc Trnt1 Fyn Src Sars2 Centg3 Map3k3 Opa1 Jak2 Rad51 Myh10 Kif19a Map2k4 Gtf2f1 Aurka Prkcd Hspa5 Ephb2 Epb4 Drg2 Gtpbp2 Fgfr2 Pcsk5 Tek Mast2 Grpel1 Rarsl Arfrp1 Eif2b1 Cabc1 Srpr Pgk1 Bub1 Pex6 E130016E03Rik Atp8b1 2610019A05Rik Rars Rac1 Bub1b Atp2a2 Skiv2l2 Xrcc5 Eif2b2 Mat2a Dek Map4k5 Atp5a1 Dock7 Actb Rab22a Recc1 Eif5 Paics Ruvbl1 Kif2c Tgm2 Sars Trap1 Arl5c Axl Farsla Top2a 2310057M21Rik Trio Hsp90b1 Myo5b Hspa8 Ckmt1

[translation factor activity, nucleic acid binding (GO:0008135)](http://amigo.geneontology.org/cgi-bin/amigo/go.cgi?view=assoc&search_constraint=terms&query=GO:0008135)

Eif4g1 3300001M20Rik Etf1 Pcbp1 Gtf2f1 Gtpbp2 Eif3s4 Eif5a Pet112l Eif2b1 Lgtn Eif2b2 Eif2s1 Eif5 Gfm2 Guf1 Eif3s7 Lama5 AU014645 2010003J03Rik Eif4a1 Bzw2 Phf3 Eef1a1 Eef1d Eif2ak1 Emg1 Hbs1l Eif2b4 Eif3s2 Mtif2 Tceb3 Eif1a Eif5b Gspt1 Eif3s1 Eif2s2 Eif1

[cytoskeletal protein binding (GO:0008092)](http://amigo.geneontology.org/cgi-bin/amigo/go.cgi?view=assoc&search_constraint=terms&query=GO:0008092)

Gabarapl2 Arl8b Nf2 Fyn Myh10 Sdc2 Epb4 Dmd Gsn Mast2 Eps8l2 Dstn Ppp1r9b Utrn Actn3 Tpm1 Ywhag Stmn1 AI428936 Myo5b Phactr1 Plec1 Farp2 Lmo7 Tpm4 Kif1b Sdc1 Marcks Egfr Actn2 Spna2 Myh3 Mtap4 Coro1b Homer2 Arpc1a Sdc4 Appbp2 Coro1c Farp1 Tmsb4x Parva Fmn2 Spnb2 Tnnt1 Jup Tmod2 Muc1 6330505N24Rik Actn1 Flnb Mlph Coro1a Myo1e Hdac6 Polb Lima1 Tln1 Lrpprc Actn4 Gmfb Tpm2 Ywhah Vcl Ktn1 Pafah1b1 Flna Fxyd5

[receptor binding (GO:0005102)](http://amigo.geneontology.org/cgi-bin/amigo/go.cgi?view=assoc&search_constraint=terms&query=GO:0005102)

Cd8b1 Glg1 Bmp1 Csh1 Csf1 Wnt7a Pthlh Tmpo Itga5 Sst Il12b Actn4 Gmfb Wnt9b Ywhah Adrb3 Fgf20 Derl1 Ccr2 4833420G17Rik Sct Fgf14 Ereg Rps2 D230025D16Rik 2010204N08Rik Prlpl Ccl19 Igf2 Itgb5 Ccr10 Il1a Lrpap1 Ephb2 Mfge8 Ptch1 Itgb1 Inhbb Bmp7 Actn3 Ywhag Adam10 Nrg4 Spp1 Spred1 Ctnnb1 Trap1 Hsp90b1 Fgf13 Il4 Il18 Ccl25 Gast Mecr Lama5 Il1rn Nsmaf Dtprp Icosl S100a6 Nisch Bcap31 Fga Hgf Ccne1 Inhba Tollip Fgf8 Iapp Dkk1 Actn2 Tnc Cdk7 D6Wsu176e Ccl5 Rb1 Fgf11 Psmc5 Dll4 Tdgf1 Wnt6 Cd40lg Timp2 Ncor2 Fgf12 Vegfc Ccl24 Gdf5 Grb2 Ppy Ik Arpp19 Cxcl12 Strap Grem2 Tac1 Wnt10a Sema3c Actn1 Ncoa1 Trip4 Shh Adm Hdgf Nrip1 F2r Jag2

[unfolded protein binding (GO:0051082)](http://amigo.geneontology.org/cgi-bin/amigo/go.cgi?view=assoc&search_constraint=terms&query=GO:0051082)

Trap1 Hsp90b1 Hspa8 Pdrg1 Ppia Hspd1 Ube4b Canx Cct5 Dnajc5 Hspe1 Tcp1 Calr Dnaja3 LOC434860 Ttc1 Cct4 Cct7 Ugcgl1 Pfdn5 Lrpap1 Hspa5 Grpel1

[identical protein binding (GO:0042802)](http://amigo.geneontology.org/cgi-bin/amigo/go.cgi?view=assoc&search_constraint=terms&query=GO:0042802)

Tcf2 Amotl2 1200015A19Rik Grpel1 Inhbb Cebpg Actn3 Ywhag Adam10 Casc3 Adipor2 Exosc8 Paics Alad 2310057M21Rik Ptpre Bhlhb8 S100a6 Timeless S100a1 Cldn1 Inhba Egfr Actn2 Robo1 Rpp14 Foxp2 Tpd52l2 Bnip3 Cldn4 Acy3 Eif2ak1 Stk25 Trip13 Phr1 Itga2 Was Actn1 Sufu Foxp1 Rassf1 Cln6 Cldn3 1200014M14Rik Cst3 Cct7 Il12b Actn4 Adrb3

[transferase activity, transferring phosphorus-containing groups (GO:0016772)](http://amigo.geneontology.org/cgi-bin/amigo/go.cgi?view=assoc&search_constraint=terms&query=GO:0016772)

Map4k5 Zc3hc1 Axl Trio Pole3 Ckmt1 Ccnb1 Hipk3 Cdk4 Nek8 Ccrk Dcakd Speg Ankk1 Pfkm Plk1 Prkcn Camk2g Acvr1b Oxsr1 Melk Pdgfrb Cdc42bpa Fgfr1 Epha4 Prkcz Trib3 Pck2 Ttn Cdkn2c Ccne1 Prkcbp1 Egfr Cds2 Sh3kbp1 Cdk7 Psmc5 Dbf4 Dgkg Csnk1d Insr Lmtk2 Neo1 Cdkn1b Dgkb Pole2 Ak3l1 Surb7 Hif1a Fgfr4 Pfkl Ccnd1 Tep1 Eif2ak1 Pank3 Stk25 Ern1 Pnpt1 Csnk2a2 Strap Pcna Twistnb Bmpr1b Pkm2 Stk3 Cdkn2b Acvrl1 BC023882 Plau Ak2 Cdk6 Met Riok2 Nr2c2 Ihpk1 Polr2g Papd1 Tgfbr2 Cdkn1a C230081A13Rik Ulk1 Ccnh Nrp1 Polb Mobkl1a Tyro3 Sphk2 Mlf1ip Limk2 Dyrk1a Uck2 Pank2 Stk24 Map2k1 Mapk8 Sbk1 Epha2 Itpk1 Pink1 Bmpr1a Prkar2b Stk38 Adpgk Prim1 Pskh1 Ephb6 Ptk2 Nadk Riok1 Tbrg4 Stk39 Mapk14 Polr3c Pdk1 Trnt1 Pip5k2c Fyn 2900024C23Rik Src Map3k3 Jak2 Brd2 Map2k4 Ubox5 Gtf2f1 Aurka Prkcd Ephb2 Fgfr2 Tek Mast2 Trrap BC068171 Cabc1 Pgk1 Bub1 Polr1e 2610019A05Rik Phka2 Bub1b Usp14 Polr3f Wdr62 Polr2i

[RNA binding (GO:0003723)](http://amigo.geneontology.org/cgi-bin/amigo/go.cgi?view=assoc&search_constraint=terms&query=GO:0003723)

Ddx55 Prkra Cirbp Polr2g Mrpl3 Papd1 Secisbp2 Hdlbp Kars Ddx4 Igf2bp1 Eif1a Rpl7 Mki67ip Syncrip Pabpc1 Nip7 Fbl Rnaset2 Tlr7 Rabgap1 Rbm18 Rad21 Ncl Sip1 Rps2 Pabpc4 Eif4g1 Csdc2 Trnt1 D330037H05Rik Pcbp1 Mrpl21 Rbm28 Snrpa1 D19Bwg1357e Eif3s4 Lgtn BC068171 Srpr Exosc1 Cugbp2 Dscr1l2 Auh Dek Exosc8 Eif2s1 Tsn Smndc1 Rbm3 Igf2bp3 Bicc1 U2af1l4 Dlx2 AU014645 Exosc4 0610009D07Rik Nkrf 2010003J03Rik Eif4a1 Ddx56 Nxf1 Magoh Rps24 Rg9mtd1 Ppil4 Nol5a Cpsf3 Ddx52 Rpp14 Sfrs7 Oas1c Sfrs10 Rnmtl1 Ddx18 Ptrf Dhx8 Rpl32 Pa2g4 Zfp36l1 Prpf4 BC003993 Tep1 Ddx41 2610033H07Rik Rps27 Sfrs3 Cpeb2 Pnpt1 Ybx1

[ligase activity, forming carbon-oxygen bonds (GO:0016875)](http://amigo.geneontology.org/cgi-bin/amigo/go.cgi?view=assoc&search_constraint=terms&query=GO:0016875)

Sars Farsla Tarsl1 Gars Aars Ptov1 Wars Kars Cdh16 Lamp2 Iars Farslb Sars2 Rarsl Hars2 Rars

[transcription factor binding (GO:0008134)](http://amigo.geneontology.org/cgi-bin/amigo/go.cgi?view=assoc&search_constraint=terms&query=GO:0008134)

Thrap3 Pfdn5 Trrap Mxd4 Cebpg Rnf12 Mbd1 Ctnnb1 Aes Crsp8 Hipk3 Hdac3 Drg1 Smarca4 Atf2 Trib3 Creb1 Ccne1 Cdk7 Rb1 Psmc5 Mtf1 Tcf20 Ecd Smarcc1 Ncor2 Myst4 Pdlim1 Trip13 LOC434860 Ncoa1 Rabl3 Trip4 Tle1 Prrx1 Sin3a Sertad2 Apex1 Nrip1 Hdac5 Sufu Wwtr1 Gli2 Stat3 Rybp Rxra Btg1 Mef2d Crsp9 Atf7ip

[hydrolase activity, acting on acid anhydrides (GO:0016817)](http://amigo.geneontology.org/cgi-bin/amigo/go.cgi?view=assoc&search_constraint=terms&query=GO:0016817)

Atp5a1 Rab22a Recc1 Ruvbl1 2310057M21Rik Hspa8 Nras Gfm2 Bcs1l Guf1 Orc4l Gbp2 Nav3 Kras Rab7l1 Cntn4 Kif1b Atp1b3 Dync1h1 Smarca4 Eif4a1 Ddx56 Atp6v0b Ddx31 Abcd2 Psmc5 Ddx52 Rhoa Eef1a1 Ddx18 Dhx8 Tubb2c Ddx20 Nsf D6Wsu116e Psmc1 1300010F03Rik Ddx39 Mov10 Ddx41 Atp5o Hbs1l Trip13 Ddx55 Fignl1 Mtif2 Cetn2 Rab3d Atp5f1 Atp1a1 Tubb3 Atad3a Atp11a Gnai1 Rala Orc1l Rab13 Dhx29 Ddx4 Rab5a Tubg2 Eif5b Atp6v1a Atp5b Rab14 Abce1 Atp1b1 Gspt1 Mapk8 Mcm5 Atf7ip Abcc3 Arl8b Abcb6 Psmc3 Rragc Opa1 Rad51 Myh10 Gtpbp2 Arfrp1 Srpr Pex6 Atp8b1 Rac1 Atp2a2 Skiv2l2 Ipo8 Xrcc5

[protein dimerization activity (GO:0046983)](http://amigo.geneontology.org/cgi-bin/amigo/go.cgi?view=assoc&search_constraint=terms&query=GO:0046983)

Ptpre Bhlhb8 S100a6 Timeless S100a1 Atf2 Creb1 Pbx1 Egfr Actn2 Robo1 Tef Ppp2ca Foxp2 Tpd52l2 Bnip3 Hif1a Hexa Eif2ak1 Phr1 Stk3 Actn1 Foxp1 Cln6 Atf4 Stat3 Limk2 Cst3 Il12b Actn4 Adrb3 Bach1 Tcf2 Timp4 Ereg Rragc Grpel1 Inhbb Cebpg Actn3 Ywhag Adam10 Jun Adipor2

[adenyl nucleotide binding (GO:0030554)](http://amigo.geneontology.org/cgi-bin/amigo/go.cgi?view=assoc&search_constraint=terms&query=GO:0030554)

Myh3 Abcd2 Cdk7 Psmc5 Ddx52 Hspe1 Aars Ptov1 Csnk1d Oas1c Insr Lmtk2 Itm2c Neo1 Supv3l1 Ddx18 Ak3l1 Dhx8 Tcp1 Ddx20 Fgfr4 Nsf Psmc1 1300010F03Rik Ddx39 Pfkl Mov10 Tep1 Ddx41 Eif2ak1 Pank3 Stk25 Ern1 Csnk2a2 Wars Trip13 Ddx55 Bmpr1b Fignl1 Stk3 Cetn2 Chdh Acvrl1 Cct4 Rabl3 Ak2 Cdk6 Gss Met Riok2 Nr2c2 Atp1a1 Atad3a Myo1e Atp11a Itm2b Tgfbr2 Orc1l C230081A13Rik Ulk1 Glud1 Kars Gtf2f2 Dhx29 Ddx4 Cdh16 Pcsk6 Acox1 Tyro3 Txnrd1 Chd8 Sphk2 Atp6v1a Atp5b Limk2 Myo1a Dyrk1a Uck2 Pank2 Stk24 Cct7 Abce1 Map2k1 Lamp2 Mapk8 Actg1 Sbk1 Smarca2 Epha2 Itpk1 Iars Mcm5 Pink1 Bmpr1a Prkar2b Dld Stk38 Hspa14 Ppox Timp4 Farslb Acads Pskh1 Ephb6 Ptk2 Gabarapl2 Gsr Thrap3 Abcc3 Riok1 Tbrg4 Stk39 Mapk14 Abcb6 Psmc3 Pdk1 Kifc3 Trnt1 Fyn Src Sars2 Map3k3 Jak2 Rad51 Myh10 Kif19a Map2k4 Gtf2f1 Aurka Prkcd Hspa5 Ephb2 Epb4 Fgfr2 Pcsk5 Tek Mast2 Grpel1 Rarsl Cabc1 Pgk1 Bub1 Pex6 E130016E03Rik Atp8b1 2610019A05Rik Rars Bub1b Atp2a2 Skiv2l2 Xrcc5 Eif2b2 Mat2a Map4k5 Atp5a1 Actb Recc1 Paics Ruvbl1 Kif2c Sars Trap1 Axl Farsla Top2a 2310057M21Rik Trio Hsp90b1 Myo5b Hspa8 Ckmt1 Bcs1l Hipk3 Cdk4 Nek8 Orc4l Ccrk Dcakd Speg Nav3 Kifc2 Kif27 Ankk1 Pfkm Plk1 Mod1 Hspd1 Prkcn Camk2g Acvr1b Inoc1 Cntn4 Kif1b Oxsr1 Ankrd17 Hspa4 Melk Pdgfrb Cdc42bpa Dync1h1 Smarca4 Fgfr1 Epha4 Prkcz Eif4a1 Trib3 Ddx56 Tarsl1 Ttn Gars Egfr Cct5 Ddx31

[translation initiation factor activity (GO:0003743)](http://amigo.geneontology.org/cgi-bin/amigo/go.cgi?view=assoc&search_constraint=terms&query=GO:0003743)

Eif2b4 Eif3s2 Mtif2 Eif1a Eif5b Eif3s1 Eif2s2 Eif1 Eif4g1 3300001M20Rik Gtf2f1 Eif3s4 Eif5a Eif2b1 Lgtn Eif2b2 Eif2s1 Eif5 Guf1 Eif3s7 Lama5 AU014645 2010003J03Rik Eif4a1 Bzw2 Eif2ak1

[actin binding (GO:0003779)](http://amigo.geneontology.org/cgi-bin/amigo/go.cgi?view=assoc&search_constraint=terms&query=GO:0003779)

Tmod2 Muc1 6330505N24Rik Actn1 Flnb Mlph Coro1a Myo1e Hdac6 Lima1 Tln1 Actn4 Gmfb Tpm2 Ywhah Vcl Flna Fxyd5 Gabarapl2 Myh10 Epb4 Dmd Gsn Eps8l2 Dstn Ppp1r9b Utrn Actn3 Tpm1 Ywhag AI428936 Myo5b Phactr1 Plec1 Lmo7 Tpm4 Marcks Egfr Actn2 Spna2 Myh3 Coro1b Homer2 Arpc1a Coro1c Tmsb4x Parva Fmn2 Spnb2

[transcription cofactor activity (GO:0003712)](http://amigo.geneontology.org/cgi-bin/amigo/go.cgi?view=assoc&search_constraint=terms&query=GO:0003712)

Trip13 LOC434860 Ncoa1 Trip4 Tle1 Prrx1 Sin3a Sertad2 Apex1 Nrip1 Hdac5 Sufu Wwtr1 Gli2 Rybp Rxra Btg1 Mef2d Crsp9 Atf7ip Thrap3 Pfdn5 Trrap Mxd4 Rnf12 Mbd1 Ctnnb1 Aes Crsp8 Hipk3 Atf2 Trib3 Creb1 Ccne1 Cdk7 Rb1 Psmc5 Mtf1 Tcf20 Ecd Smarcc1 Ncor2 Pdlim1

[integrin binding (GO:0005178)](http://amigo.geneontology.org/cgi-bin/amigo/go.cgi?view=assoc&search_constraint=terms&query=GO:0005178)

Itga5 Actn4 Itgb5 Mfge8 Itgb1 Actn3 Adam10 Spp1 Lama5 Nisch Actn2 Timp2 Actn1

[phosphotransferase activity, alcohol group as acceptor (GO:0016773)](http://amigo.geneontology.org/cgi-bin/amigo/go.cgi?view=assoc&search_constraint=terms&query=GO:0016773)

Map4k5 Axl Trio Hipk3 Cdk4 Nek8 Ccrk Dcakd Speg Ankk1 Pfkm Plk1 Prkcn Camk2g Acvr1b Oxsr1 Melk Pdgfrb Cdc42bpa Fgfr1 Epha4 Prkcz Trib3 Ttn Egfr Cdk7 Dbf4 Dgkg Csnk1d Insr Lmtk2 Neo1 Dgkb Hif1a Fgfr4 Pfkl Ccnd1 Eif2ak1 Pank3 Stk25 Ern1 Csnk2a2 Bmpr1b Pkm2 Stk3 Acvrl1 Cdk6 Met Riok2 Nr2c2 Ihpk1 Tgfbr2 Cdkn1a C230081A13Rik Ulk1 Ccnh Nrp1 Tyro3 Sphk2 Limk2 Dyrk1a Pank2 Stk24 Map2k1 Mapk8 Sbk1 Epha2 Itpk1 Pink1 Bmpr1a Prkar2b Stk38 Adpgk Pskh1 Ephb6 Ptk2 Nadk Riok1 Tbrg4 Stk39 Mapk14 Pdk1 Pip5k2c Fyn Src Map3k3 Jak2 Brd2 Map2k4 Gtf2f1 Aurka Prkcd Ephb2 Fgfr2 Tek Mast2 Trrap Pgk1 Bub1 2610019A05Rik Phka2 Bub1b

[hydrolase activity, acting on acid anhydrides, in phosphorus-containing anhydrides (GO:0016818)](http://amigo.geneontology.org/cgi-bin/amigo/go.cgi?view=assoc&search_constraint=terms&query=GO:0016818)

Atp5a1 Rab22a Recc1 Ruvbl1 2310057M21Rik Hspa8 Nras Gfm2 Bcs1l Guf1 Orc4l Gbp2 Nav3 Kras Rab7l1 Cntn4 Kif1b Atp1b3 Dync1h1 Smarca4 Eif4a1 Ddx56 Atp6v0b Ddx31 Abcd2 Psmc5 Ddx52 Rhoa Eef1a1 Ddx18 Dhx8 Tubb2c Ddx20 Nsf D6Wsu116e Psmc1 1300010F03Rik Ddx39 Mov10 Ddx41 Atp5o Hbs1l Trip13 Ddx55 Fignl1 Mtif2 Cetn2 Rab3d Atp5f1 Atp1a1 Tubb3 Atad3a Atp11a Gnai1 Rala Orc1l Rab13 Dhx29 Ddx4 Rab5a Tubg2 Eif5b Atp6v1a Atp5b Rab14 Abce1 Atp1b1 Gspt1 Mapk8 Mcm5 Atf7ip Abcc3 Arl8b Abcb6 Psmc3 Rragc Opa1 Rad51 Myh10 Gtpbp2 Arfrp1 Srpr Pex6 Atp8b1 Rac1 Atp2a2 Skiv2l2 Ipo8 Xrcc5

Under represented

[receptor activity (GO:0004872)](http://amigo.geneontology.org/cgi-bin/amigo/go.cgi?view=assoc&search_constraint=terms&query=GO:0004872)

Ncoa1 Trip4 Ptprg Met Nrip1 Nr2c2 Il17ra F2r Hyal2 Ptgfrn Dag1 Ptprs Cd86 Cd8b1 Tgfbr2 Nrp1 Mtus1 Ccr6 Pscd3 Tyro3 Sfrp4 Cldn3 Tlr7 Itga5 Ptpn13 Rxra Adrb3 Tfrc Olfm1 Derl1 Epha2 Ccr2 Bmpr1a Plxna3 Ntsr1 Itga1 Sv2c Crsp9 Ephb6 Thrap3 Lrp2 Plxna4 Itgb5 Ccr10 Itga8 Procr Lrpap1 Ephb2 Eps8 Fgfr2 Tek Itga9 Ptch1 Lgtn Srpr Itgb1 Il10ra Ptprn Utrn Tm2d1 Aven Glp1r Slc20a1 Nr5a2 Cd14 Lsr Adipor2 C1qtnf5 Igf2r Plxna2 Edg5 Cfi Tlr2 Trap1 Axl Unc5b Ccr8 Ptpre Thrap2 Lrp1 Lama5 Il1rn Ssr1 Notch3 Itga7 H47 Acvr1b Bcap31 Cntn4 Lrp8 Eps15 Pdgfrb Fgfr1 Epha4 Ptpra Plxnb2 Fzd7 Pigr Efemp2 Ttn Cul5 Egfr Itga3 Ptpn11 Dmbt1 Gprc5a Synj2bp Robo1 F3 Pgrmc2 Trfr2 Tnfrsf19 Insr Tgfbr3 Neo1 Il8rb Hnf4a Lphn2 Ncor2 Atrnl1 Fgfr4 Grb2 Tmem11 Med6 Fzd5 Adam17 Gpc1 Smo Tomm22 Fcgrt Strap Trip13 Ccr3 Vldlr Bmpr1b Cd44 Ptprcap Vcam1 Fzd1 Edg2 Acvrl1 Itga2

[channel or pore class transporter activity (GO:0015267)](http://amigo.geneontology.org/cgi-bin/amigo/go.cgi?view=assoc&search_constraint=terms&query=GO:0015267)

Nup155 Aven Kctd5 Vdac3 Nup37 Nup88 Clcn5 Nup43 Nup153 Kcnab1 Klhl24 Fxyd5 Kctd2

[ion transporter activity (GO:0015075)](http://amigo.geneontology.org/cgi-bin/amigo/go.cgi?view=assoc&search_constraint=terms&query=GO:0015075)

Cox6b1 Atp8b1 Aven Slc6a6 Atp2a2 Slc11a2 Kctd5 Atp5a1 Slc30a2 Slc39a8 Atp1b3 Slc30a9 Atp6v0b Tmco3 Uqcrb Slc12a7 Tspan5 Ank Clcn5 Slc6a8 Atp5o Hiatl1 Atp5f1 Atp1a1 Cox10 Atp11a Slc12a6 Slc28a2 Slc4a2 Cox4i1 Atp6v1a Atp5b Atp1b1 Mapk8 Kcnab1 Tfrc Klhl24 Fxyd5 Kctd2 Slc31a2

[neurotransmitter binding (GO:0042165)](http://amigo.geneontology.org/cgi-bin/amigo/go.cgi?view=assoc&search_constraint=terms&query=GO:0042165)

Ntsr1 Ccr3 Adrb3

[transmembrane receptor activity (GO:0004888)](http://amigo.geneontology.org/cgi-bin/amigo/go.cgi?view=assoc&search_constraint=terms&query=GO:0004888)

Met Il17ra F2r Dag1 Nrp1 Ccr6 Sfrp4 Cldn3 Tlr7 Adrb3 Tfrc Olfm1 Epha2 Ccr2 Bmpr1a Plxna3 Ntsr1 Ephb6 Ccr10 Lrpap1 Ephb2 Fgfr2 Ptch1 Il10ra Utrn Tm2d1 Glp1r Lsr C1qtnf5 Edg5 Cfi Tlr2 Ccr8 Acvr1b Lrp8 Pdgfrb Fgfr1 Epha4 Fzd7 Efemp2 Ttn Egfr Dmbt1 Gprc5a F3 Trfr2 Tnfrsf19 Insr Neo1 Il8rb Lphn2 Fgfr4 Tmem11 Fzd5 Gpc1 Smo Ccr3 Vldlr Bmpr1b Fzd1 Edg2 Acvrl1

[alpha-type channel activity (GO:0015268)](http://amigo.geneontology.org/cgi-bin/amigo/go.cgi?view=assoc&search_constraint=terms&query=GO:0015268)

Aven Kctd5 Clcn5 Kcnab1 Klhl24 Fxyd5 Kctd2
